# Does Ortho-Substitution Enhance Cytotoxic Potencies in a Series of 3,5-Bis(benzylidene)-4-piperidones?

**DOI:** 10.3390/medicines11080019

**Published:** 2024-10-30

**Authors:** Subhas S. Karki, Umashankar Das, Jan Balzarini, Erik De Clercq, Hiroshi Sakagami, Yoshihiro Uesawa, Praveen K. Roayapalley, Jonathan R. Dimmock

**Affiliations:** 1 College of Pharmacy and Nutrition, University of Saskatchewan, Saskatoon, SK S7N 5C9, Canada; subhasskarki@gmail.com (S.S.K.); jr.dimmock@usask.ca (J.R.D.); 2 Department of Pharmaceutical Chemistry, KLE College of Pharmacy-Bengaluru (A Constituent Unit of KAHER-Belagavi), Bengaluru 560010, Karnataka, India; 3 Rega Institute of Medical Research, Katholieke Universiteit Leuven, 3000 Leuven, Belgium; 4 Mekei University Research Institute of Odontology (M-RIO), School of Dentistry, Meikai University, Sakado 350-0238, Saitama, Japan; sakagami@dent.meikai.ac.jp; 5 Department of Medical Molecular Informatics, Meiji Pharmaceutical University, Tokyo 204-858, Japan; uesawa@my-pharm.ac.jp

**Keywords:** 4-piperidones, conjugated unsaturated ketones, ortho-effect, cytotoxicity, tumor-selective toxicity, QSAR, SAR, molecular modeling

## Abstract

**Background:** A series of 3,5-benzylidene-4-piperidones, **1a**–**n**, were prepared to evaluate the hypothesis that the placement of different groups in the ortho-location of the aryl rings led to compounds with greater cytotoxic potencies than structural analogs. **Methods:** The bioevaluation of **1a**–**n** was undertaken using human Molt/4C8 and CEM cells as well as murine L1210 cells. Correlations were sought between the interplanar angles θ_A_ and θ_B_ and the cytotoxic potencies. A QSAR analysis was also undertaken. In order to evaluate whether these compounds demonstrated greater toxicity to neoplasms than non-malignant cells, **1a**–**n** were evaluated against HSC-2, HSC-3, HSC-4 and HL60 neoplasms as well as non-malignant HGF, HPC and HPLF cells. **Results:** A positive correlation was noted between the interplanar angle θ_A_ of one of the aryl rings and the adjacent olefinic linkage with IC_50_ values in the Molt4/C8 screens. The QSAR analysis revealed a positive correlation between the Hansch pi (π) value of the aryl substituents and the IC_50_ values of the compounds towards the Molt4/C8 and CEM cells. The dienones in series **1** demonstrated higher tumor-selective toxicity towards HSC-2, HSC-3, HSC-4 and HL-60 neoplasms than HGF, HPC and HPLF cells. **Conclusions:** The bioevaluations revealed some support for greater cytotoxic potencies to be displayed by compounds having ortho-substituents.

## 1. Introduction

For a number of years, the principal focus in our laboratories has been the design, syntheses and antineoplastic evaluation of conjugated enones. These compounds interact with thiols but are far less reactive towards amino and hydroxyl groups, which are found in nucleic acids [1,2,3]. Hence, the problem of genotoxicity found in a number of contemporary anticancer drugs [4] may be avoided by the use of these compounds. In recent times, the emphasis has focused on the development of 1,5-diaryl-3-oxo-1,4-pentadienes [ARCH=C(R)-C-(O)-C(R)=CHAR], which permit sequential interactions with cellular thiols. Successive chemical attacks have been shown on occasions to be more toxic to neoplasms than non-malignant cells [5,6]. In fact, a greater toxicity to cancers than normal cells has been observed with various conjugated unsaturated ketones [7].

A number of studies have revealed the potent cytotoxicity of compounds, in which the 1,5-diaryl-3-oxo-1,4-pentadienyl group was mounted on a piperidyl scaffold [8,9,10]. In these investigations, the substituents were located in the meta- and para-positions of the aryl rings for a number of reasons, including seeking correlations between the magnitude of the Hammett (σ) values and the cytotoxic properties.

A major emphasis in the present investigation is to ascertain whether ortho-substitution enhances cytotoxic potencies compared to the placement of the same groups in the meta- and para-locations. The other goals of this investigation are to find potent antineoplastic agents serving as cytotoxic warheads which demonstrate a greater toxicity to tumors than non-malignant cells. Should these compounds appear to be promising, then quests to determine the structure–activity relationships (SARs) and quantitative structure–activity relationships (QSARs) will be undertaken.

In this study, an ortho-effect is defined as the contribution to cytotoxic potencies by introducing a substituent into the ortho-position of an aryl ring. In particular, comparisons are made between the cytotoxic potencies of an ortho-substituted compound with the analogs in which the same substituent is placed in the meta- and para-positions of the aryl ring.

## 2. Materials and Methods

The melting points were uncorrected. The ^1^H-NMR spectra were recorded using a Bruker Avance AMX 500 FT spectrometer (Billerica, MA, USA) equipped with a BBO probe in Supplementary Materials. The chemical shifts are reported in ppm. Elemental analyses were obtained by using an Elementar CHNS analyzer (Langenselbold, Hesse, Germany).

### 2.1. Synthesis of Compounds

Dry hydrogen chloride gas was passed to a stirring suspension of 4-piperidone hydrochloride monohydrate (0.003 mol) and aryl aldehyde (0.006 mol) in glacial acetic acid (50 mL) until a clear solution was obtained. The reaction mixture was stirred at room temperature overnight. The solid thus formed was filtered and washed with acetone to remove the residual acetic acid and aldehyde. The solid was neutralized with aqueous potassium carbonate solution (15 mL, 10% *w*/*v*), stirred at room temperature for 1 h, filtered, dried and recrystallized from ethanol to provide the pure compounds. The percentage yield, melting points, ^1^H-NMR spectra and elemental analyses of **1a**–**n** are presented as follows.

**3,5-bis(2-Fluorobenzylidene)-4-piperidone (1a)**

Yield: 45%. Mp: 138–140 °C. ^1^H NMR (500 MHz, DMSO-d_6_): δ 7.66 (s, 2H, olefinic), 7.52–7.46 (m, 4H, Ar), 7.36–7.30 (m, 4H, Ar) and 3.91 (s, 4H, pip H). Calcd for C_19_H_15_F_2_NO: 0.5 H_2_O: C, 71.17%; H, 4.99%; N, 4.37%. Found: C, 71.53%; H, 4.78%; N, 4.35%.

**3,5-bis(3-Fluorobenzylidene)-4-piperidone (1b)**

Yield: 50%. Mp: 195–198 °C. ^1^H NMR (500 MHz, DMSO-d_6_): δ 7.75 (s, 2H, olefinic), 7.59–7.54 (m, 2H, Ar), 7.42–7.31 (m, 6H, Ar) and 4.29 (s, 4H, pip H). Calcd for C_19_H_15_F_2_NO: 0.25 H_2_O: C, 72.19%; H, 4.90%; N, 4.43%. Found: C, 72.15%; H, 4.74%; N, 4.43%.

**3,5-bis(2,4-Difluorobenzylidene)-4-piperidone (1c)**

Yield: 45%. Mp: 182–185 °C. ^1^H NMR (500 MHz, DMSO-d_6_): δ 7.59 (s, 2H, olefinic), 7.56–7.51 (m, 2H, Ar), 7.44–7.40 (m, 2H, Ar), 7.23–7.20 (t, 2H, J = 16.8 Hz, Ar) and 3.89 (s, 4H, pip H). Calcd for C_19_H_13_F_4_NO: C, 65.71%; H, 3.77%; N, 4.03%. Found: C, 65.76%; H, 3.61%; N, 3.95%.

**3,5-bis(2,5-Difluorobenzylidene)-4-piperidone (1d)**

Yield: 45%. Mp: 192–195 °C. ^1^H NMR (500 MHz, DMSO-d_6_): δ 7.57 (s, 2H, olefinic), 7.43–7.32 (m, 6H, Ar) and 3.91 (s, 4H, pip H). Calcd for C_19_H_13_F_4_NO: C, 65.71%; H, 3.77%; N, 4.03%. Found: C, 65.67%; H, 3.80%; N, 3.77%.

**3,5-bis(2,6-Difluorobenzylidene)-4-piperidone (1e)**

Yield: 60%. Mp: 194–195 °C. ^1^H NMR (500 MHz, DMSO-d_6_): δ 7.59–7.53 (m, 2H, Ar), 7.39 (s, 2H, olefinic), 7.26–7.23 (t, 4H, J = 16.2 Hz, Ar) and 3.62 (s, 4H, pip H). Calcd for C_19_H_13_F_4_NO: C, 65.71%; H, 3.77%; N, 4.03%. Found: C, 65.57%; H, 3.47%; N, 3.91%.

**3,5-bis(2-Chlorobenzylidene)-4-piperidone (1f)**

Yield: 40%. Mp: 144–148 °C. ^1^H NMR (500 MHz, DMSO-d_6_): δ 7.75 (s, 2H, olefinic), 7.60–7.59 (m, 2H, Ar), 7.48–7.45 (m, 6H, Ar) and 3.89 (s, 4H, pip H). Calcd for C_19_H_15_Cl_2_NO: C, 66.29%; H, 4.39%; N, 4.07%. Found: C, 66.16%; H, 4.16%; N, 3.88%.

**3,5-bis(2,4-Dichlorobenzylidene)-4-piperidone (1g)**

Yield: 55%. Mp: 180–184 °C. ^1^H NMR (500 MHz, DMSO-d_6_): δ 7.79–7.78 (m, 2H, Ar), 7.68 (s, 2H, olefinic), 7.54–7.52 (m, 2H, Ar), 7.48–7.46 (m, 2H, Ar) and 3.87 (s, 4H, pip H). Calcd for C_19_H_13_Cl_4_NO: C, 55.24%; H, 3.17%; N, 3.39%. Found: C, 55.27%; H, 3.07%; N, 3.25%.

**3,5-bis(2,6-Dichlorobenzylidene)-4-piperidone (1h)**

Yield: 50%. Mp: 183–185 °C. ^1^H NMR (500 MHz, DMSO-d_6_): δ 7.60–7.59 (m, 4H, olefinic, Ar), 7.48–7.45 (m, 4H, Ar) and 3.50 (s, 4H, pip H). C_19_H_13_Cl_4_NO: C, 55.24%; H, 3.17%; N, 3.39%. Found: C, 55.29%; H, 3.02%; N, 3.29%.

**3,5-bis(2-Bromobenzylidene)-4-piperidone (1i)**

Yield: 60%. Mp: 168–170 °C. ^1^H NMR (500 MHz, DMSO-d_6_): δ 7.78 (d, 2H, J = 8.2 Hz, Ar), 7.70 (s, 2H, olefinic), 7.51–7.48 (m, 2H, Ar), 7.44–7.43 (d, 2H, J = 6.6 Hz, Ar), 7.39–7.36 (m, 2H, Ar) and 3.87 (s, 4H, pip H). Calcd for C_19_H_15_Br_2_NO: C, 52.69%; H, 3.49%; N, 3.23%. Found: C, 52.67%; H, 3.30%; N, 3.17%.

**3,5-bis(3-Bromobenzylidene)-4-piperidone (1j)**

Yield: 55%. Mp: 155–158 °C. ^1^H NMR (500 MHz, DMSO-d_6_): δ 7.70 (s, 2H, olefinic), 7.63 (d, 2H, J = 7.8 Hz, Ar), 7.56 (s, 2H, Ar), 7.51 (d, 2H, J = 7.7 Hz, Ar), 7.46–7.42 (m, 2H, Ar) and 3.99 (s, 4H, pip H). Calcd for C_19_H_15_Br_2_NO: C, 52.69%; H, 3.49%; N, 3.23%. Found: C, 52.70%; H, 3.41%; N, 2.96%.

**3,5-bis(2-Methylbenzylidene)-4-piperidone (1k)**

Yield: 40%. Mp: 136–139 °C. ^1^H NMR (500 MHz, DMSO-d_6_): δ 7.75 (s, 2H, olefinic), 7.31–7.22 (m, 8H, Ar), 3.85 (s, 4H, pip H) and 2.33 (s, 6H, 2-CH_3_). Calcd for C_21_H_21_NO: 0.25 H_2_O: C, 83.13%; H, 6.98%; N, 4.62%. Found: C, 82.92%; H, 7.06%; N, 4.42%.

**3,5-bis(2,4-Dimethylbenzylidene)-4-piperidone (1l)**

Yield: 30%. Mp: 153–156 °C. ^1^H NMR (500 MHz, DMSO-d_6_): δ 7.72 (s, 2H, olefinic), 7.13–7.12 (m, 4H, Ar), 7.09–7.07 (m, 2H, Ar), 3.84 (s, 4H, pip H), 2.31 (s, 6H, 2-CH_3_) and 2.29 (s, 6H, 2-CH_3_). Calcd for C_23_H_25_NO: C, 83.34%; H, 7.60%; N, 4.23%. Found: C, 82.97%; H, 7.61%; N, 4.17%

**3,5-bis(2-Methoxybenzylidene)-4-piperidone (1m)**

Yield: 55%. Mp: 158–162 °C. ^1^H NMR (500 MHz, DMSO-d_6_): δ 7.79 (s, 2H, olefinic), 7.43 (t, 2H, J = 14.9 Hz, Ar), 7.27 (d, 2H, J = 7.4 Hz, Ar), 7.11 (d, 2H, J = 8.3 Hz, Ar), 7.02 (t, 2H, J = 14.8 Hz, Ar), 3.88 (s, 4H, pip H) and 3.86 (s, 6H, 2-OCH_3_). Calcd for C_21_H_21_NO_3_: C, 75.20%; H, 6.31%; N, 4.18%. Found: C, 74.84%; H, 6.55%; N, 4.12%.

**3,5-bis(2,3-Dimethoxybenzylidene)-4-piperidone (1n)**

Yield: 40%. Mp: 199–200 °C. ^1^H NMR (500 MHz, DMSO-d_6_): δ 8.01 (s, 2H, olefinic), 7.23–7.20 (m, 4H, Ar), 6.93–6.91 (m, 2H, Ar), 4.35 (s, 4H, pip H), 3.87 (s, 6H, 2-OCH_3_) and 3.77 (s, 6H, -OCH_3_). Calcd for C_23_H_25_NO_5_: C, 69.86%; H, 6.37%; N, 3.54%. Found: C, 69.68%; H, 6.06%; N, 3.17%.

### 2.2. Cytotoxicity Assays

The evaluations of cytotoxicity of **1a**–**n** against the human leukemia T-lymphoblast cell line Molt4/C8 (accession number: CVCL_F827; database name: Cellosaurus), human T-cell lymphoma CEM (CVCL_0207) and mouse lymphocytic leukemic cells L1210 (CVCL_0382) (purchased from ATCC) in suspension culture were undertaken at 37 °C with the neoplasms for 48 h (L1210 cells) or 72 h (Molt4/C8 and CEM) in RPMI1640 medium supplemented with fetal bovine serum (FBS) [11]. In the case of **2d**, which was evaluated as hydrochloride salt, the hitherto unrecorded IC_50_ values in the Molt4/C8, CEM and L1210 bioassays were 7.70 ± 0.81, 1.70 ± 0.04 and 31.1 ± 11.0, respectively [12].

Human oral squamous cell carcinoma cell lines from tongue [HSC-2 (RCB1945), HSC-3 (RCB1975), HSC-4 (RCB1902)] and human promyelocytic leukemia HL-60 (RCB3683) were purchased from RIKEN Cell Bank, Tsukuba, Japan. Human gingival fibroblast HGF, human pulp cell HPC and human periodontal ligament fibroblast HPLF cells were prepared from the first premolar extracted tooth and periodontal tissues of a twelve-year-old girl, according to the guideline of the Institutional Board of Meikai University Ethic Committee (No. A0808)]. Human oral cells (HSC-2, HSC-3, HSC-4, HGF, HPLF and HPC) and HL-60 cells were incubated for 48 h at 37 °C with different concentrations of the dienones in DMEM or RPMI1640 media (only for HL-60 cells) (GIBCO BRL, Grand Island, NY, USA) supplemented with 10% heat-inactivated fetal bovine serum (Sigma-Aldrich Inc., St. Louis, MO, USA) and antibiotics (100 U/mL of penicillin G and 100 µg/mL of streptomycin sulfate) under a humidified 5% CO_2_ atmosphere. The viability of the attached cells (HSC-2, HSC-3, HSC-4, HGF, HPLF and HPC) was recorded using the MTT method [13]. The number of viable non-adherent cells (HL-60) was determined by a cell count with a hemocytometer after staining with 0.15% trypan blue.

### 2.3. Molecular Modeling

The interplanar angles θ_A_ and θ_B_ were obtained using a software package [14]. The equilibrium geometry of each molecule in water at the ground state was obtained using a sequence of energy minimization techniques. First, the molecules were subjected to molecular mechanics MMFF, followed by the semi-empirical PM6 and Hartree-Foch 3-21G energy minimization methods.

### 2.4. Statistical Analyses

The MR, π and σ values were obtained from the literature [15], while the σ* figures were obtained from a published source [16]. The MR value of hydrogen is 1.03, not 1.00. Hence, in order to compare the steric bulk of different groups in series **1**, the figure of 1.03 was added to the MR value of the monosubstituted compounds. For example, the MR figure of the chloro atom is 6.03, and, hence, the MR value for **1f** is 7.06 (6.03 + 1.03), and for **1g** and **1h**, the figure is 12.06 (2 × 6.03). The linear and semilogarithmic plots were made using a statistical package [17]. The calculation of the coefficient of determination (R^2^) and the significance level (*p*-value) in Pearson’s correlation analysis was performed using JMP Pro 18.0.1 (SAS Institute Inc., Cary, NC, USA). In Pearson’s correlation analysis, the CC_50_ values for Molt4/C8, CEM and L1210 were subjected to natural logarithm transformation. Each value represents the mean ± S.D. of triplicate assays.

## 3. Results

**Figure 1:**
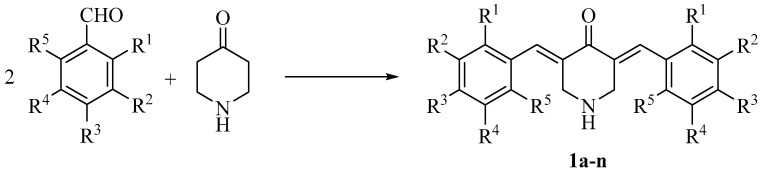
The synthesis of **1a**–**n**. The aryl substituents are presented in Table 1.

The compounds in series **1** were prepared by acid-catalyzed condensation between various substituted aryl aldehydes and 4-piperidone, as indicated in Figure 1. The evaluation of these molecules was undertaken against human Molt4/C8 and CEM T-lymphocytes as well as murine L1210 leukemic cells. All of the compounds in series **1** were also screened against human HSC-2, HSC-3 and HSC-4 oral squamous cell carcinomas as well as human HL-60 promyelocytic leukemic cells. In addition, these enones were evaluated against human HGF gingival fibroblasts, HPC pulp cells and HPLF periodontal ligament fibroblasts, which are non-malignant cells. The data generated from these determinations are displayed in in the discussion section.

## 4. Discussion

The ^1^H-NMR spectra of **1a**–**n** reveal that the olefinic protons are in the range of 7.57–8.01 ppm, indicating the *E* configuration [18]. Various substituents were placed in the aryl rings, which differed in size, electronic properties and hydrophobicity. Most of these compounds have at least one ortho-substituent.

**Table 1:** Evaluation of **1a**–**n** against Molt4/C8, CEM and L1210 cells and the interplanar angles θ_A_ and θ_B_.

Compound	Aryl Substituent	Molt4/C8 ^a^	CEM ^a^	L1210 ^a^	θ_A_ ^b^	θ_B_ ^b^
**1a**	2-F	0.94 ± 0.74	1.46 ± 0.52	7.18 ± 1.02	34.69	147.74
**1b**	3-F	0.59 ± 0.39	0.75 ± 0.42	6.48 ± 1.19	41.48	140.22
**1c**	2,4-F_2_	3.91 ± 3.15	6.21 ± 3.09	27.4 ± 17.3	34.44	148.02
**1d**	2,5-F_2_	3.26 ± 2.44	8.10 ± 0.48	37.4 ± 8.80	33.57	148.55
**1e**	2,6-F_2_	1.13 ± 0.65	3.20 ± 2.07	8.83 ± 1.80	42.51	140.17
**1f**	2-Cl	1.13 ± 0.26	1.14 ± 0.73	6.35 ± 1.63	50.64	131.52
**1g**	2,4-Cl_2_	4.70 ± 3.95	7.30 ± 0.46	34.0 ± 3.60	51.67	130.39
**1h**	2,6-Cl_2_	6.01 ± 1.46	6.51 ± 0.17	9.87 ± 1.31	78.62	103.08
**1i**	2-Br	1.33 ± 0.17	1.19 ± 0.54	2.54 ± 1.39	48.94	133.26
**1j**	3-Br	5.28 ± 2.64	7.10 ± 0.95	48.0 ± 6.70	41.65	139.96
**1k**	2-CH_3_	1.59 ± 0.07	1.62 ± 0.22	8.59 ± 0.65	51.60	130.05
**1l**	2,4-(CH_3_)_2_	5.08 ± 4.30	6.79 ± 1.66	26.0 ± 9.00	49.23	132.55
**1m**	2-OCH_3_	0.73 ± 0.44	0.91 ± 0.50	0.90 ± 0.64	−55.01	145.72
**1n**	2,3-(OCH_3_)_2_	0.36 ± 0.11	0.66 ± 0.45	0.84 ± 0.12	−56.67	144.13
**2a ^c^**	H	1.67 ± 0.15	1.70 ± 0.02	7.96 ± 0.11	39.81	141.78
**2b**	4-F	5.00 ± 1.20	2.05 ± 0.36	0.60 ± 0.01	---	---
**2c**	4-Cl	13.4 ± 4.00	8.63 ± 0.48	4.15 ± 0.30	---	---
**2d**	4-Br	7.70 ± 0.81	1.70 ± 0.04	31.1 ± 11.0	---	---
**2e**	4-CH_3_	1.69 ± 0.09	1.69 ± 0.00	8.47 ± 0.14	---	---
**2f**	4-OCH_3_	288 ± 43.0	164 ± 104	244 ± 43.0	---	---
Melphalan ^c^	----	3.24 ± 0.56	2.47 ± 0.21	2.13 ± 0.02	---	---

^a^ The figures are the IC_50_ values of the compounds which inhibit the growth of the cells by 50%. ^b^ Both aryl rings in **1m** and **1n** are orientated in the same direction. Hence, θ_A_ is determined in a counterclockwise fashion and presented as negative values. ^c^ Data taken from reference [19].

The initial bioevaluations used human Molt4/C8 and CEMT lymphocytes with a view to finding if the compounds were effective against human neoplastic cells. The data presented in Table 1 reveal, the IC_50_ values of **1a**–**n** are all below 10 µM and are in the range of 0.4–8 µM. The murine L1210 cell line was chosen because a number of anticancer drugs are cytotoxic to L1210 cells [20] and, hence, this screen may indicate one or more candidate anticancer agents. In general, the compounds are less potent towards L1210 cells than the two T-lymphocytes. Thus, the average IC_50_ values of **1a**–**n** against the Molt4/C8, CEM and L1210 cells are 2.57, 3.78 and 16.0 µM, respectively. Comparisons were made between the IC_50_ values of **1a**–**n** and the anticancer drug melphalan. In the Molt4/C8 bioassay, the following dienones are statistically significantly more potent than melphalan (fold increases in potency in parentheses), namely, **1a** (3.45), **1b** (5.49), **1e** (2.87), **1f** (2.87), **1i** (2.44), **1k** (2.04), **1m** (4.44) and **1n** (9.00). In regard to the CEM bioassay, the compounds exceeding the potency of melphalan include **1a** (1.69), **1b** (3.29), **1f** (2.17), **1i** (2.08), **1k** (1.53), **1m** (2.71) and **1n** (3.74), while **1m** and **1n** are 2.37 and 2.54 times the potency of melphalan when evaluated against L1210 cells.

**Figure 2:**
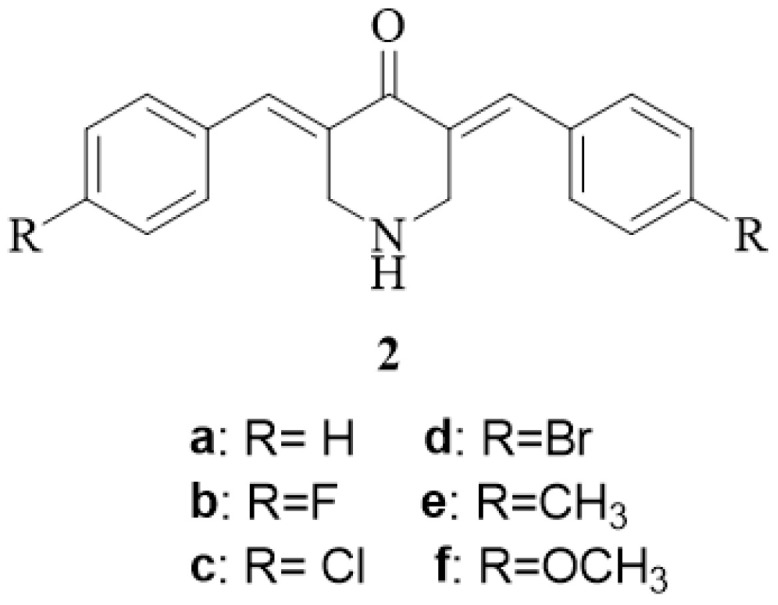
The compounds in series **2**.

Two investigations were undertaken to find whether the ortho-substitution enhanced cytotoxic potencies involving the compounds in series **1** and series **2** with substituents as indicated in Figure 2. First, comparisons were made between the potencies of **1a**,**f**,**i**,**k**,**m** and the unsubstituted compound **2a [19]**. The dienones **1f**,**i**,**m** are more potent than **2a** in the Molt4/C8 screen, while **1m** has a lower IC_50_ value than **1a** in the CEM bioassay. Both **1i** and **1m** are more potent than **2a** when evaluated against L1210 cells. Thus, in 40% of the comparisons, the insertion of an ortho-substituent into the aryl ring of **2a** gives compounds with increased potencies. In the remaining comparisons, equipotencies were noted. Second, comparisons were made between the IC_50_ values of compounds having a single ortho-substituent (**1a**,**f**,**i**,**k**,**m**) with the published figures of **2b**,**c**,**f** [19] and **2e** [21], whose potencies are reported herein. Comparisons were made between the biodata of compounds having the same aryl substituents. For example, the IC_50_ values of **1a** were compared with the IC_50_ figures generated for **2b**, **1f** with **2c**, and so forth. Cytotoxic evaluations of the dienones **1a**,**f**,**i**,**m** (Molt4/C8 screen), **1f**,**m** (CEM test) and **1a**,**f**,**i**,**k**,**m** (L1210 bioassay) reveal that, in two-thirds of the comparisons made, the placement of a substituent in the ortho-location leads to compounds with a greater potency than the structural isomer having the same group but located in the para-position. Equipotency was noted in the other comparisons made. The available evidence, therefore, is that compounds with ortho-substituents are, in general, more potent than the para-substituted analogs and, in other cases, are equipotent.

The results in Table 1 were examined with a view to determining some of the SARs, since such observations may enable the formation of guidelines for analog development. Compounds possessing a single ortho-substituent (**1a**,**f**,**i**,**k**,**m**) are promising cytotoxins having the same potencies in each of the bioassays, except for **1i** > **1a** and **1m** > **1a** in the L1210 screen. The introduction of a second aryl substituent, identical to the ortho-substituent already in the aryl rings, gave conflicting cytotoxic data. The presence of a second fluoro atom in **1c**–**e** had varying effects on the potencies. Thus, the placement of a second fluoro atom in the 4 and 5 positions of the aryl rings (**1c** and **1d**) led to increased IC_50_ values compared to **1a**. However, the 2,6-difluoro analogs **1e** and **1a** are equipotent. The addition of a second chloro atom to **1f** gave **1g**,**h**, which have higher IC_50_ figures than **1f**. The addition of a 4-methyl group to **1k** led to **1l**, which has lower potency than **1d**.

Comparisons were also made between compounds having a single ortho-substituent and the corresponding meta-structural isomer. While **1a** and **1b** are equipotent, relocating the bromo atom from the ortho-position (**1i**) to the meta-location (**1j**) led to a 4–20-fold drop in potency.

The conclusion derived from the SAR is to place other groups solely in the ortho-position of 3,5-bis(benzylidene)-4-piperidones with a view to increasing the potencies. In addition, the significant potency of **1n** suggests that a variety of 2,3-disubstituted analogs should be prepared.

**Figure 3:**
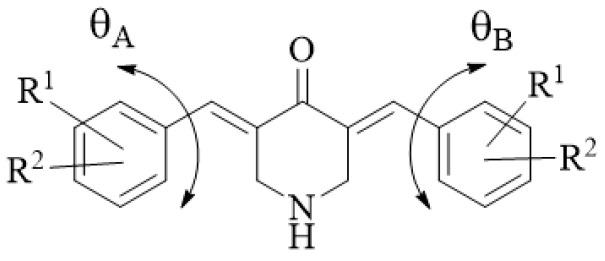
The designation of the torsion angles θ_A_ and θ_B_.

**Figure 4:**
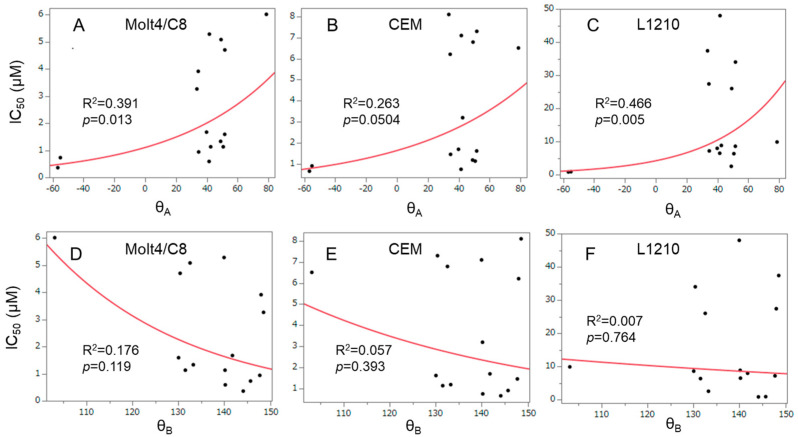
Correlation between the IC_50_ figures of **1a**–**n**/**2a** against Molt4/C8 (**A**,**D**), CEM (**B**,**E**) and L1210 (**C**,**F**) and the interplanar angles designated θ_A_ (**A**–**C**) and θ_B_ (**D**–**F**). These data are derived from Table 1.

A search was undertaken for correlations between certain physicochemical parameters and cytotoxic potencies. Substituents placed in the ortho-position of the aryl rings contribute to the lack of coplanarity between the aromatic ring and the adjacent methine group. This phenomenon gives rise to interplanar angles designated θ_A_ and θ_B_, as indicated in Figure 3. On occasions, the coplanarity, and also the lack of coplanarity, between certain functional groups of compounds have a profound effect on the bioactivities [22]. Hence, the θ_A_ and θ_B_ values of **1a**–**n** were obtained by molecular modeling, and the results are portrayed in Table 1. Positive correlations were noted between the IC_50_ figures of **1a**–**n**/**2a** in the Molt4/C8 (R^2^ = 0.391, *p* < 0.05) (A) and L1210 (R^2^ = 0.466, *p* < 0.01) (C) screens and the θ_A_ values (Figure 4). In the case of the CEM bioassay, a trend towards a positive correlation was noted (R^2^ = 0.263, *p* < 0.1) (B). Neither correlations (*p* < 0.05) nor trends to a correlation (*p* < 0.10) were found between the θ_B_ values and the cytotoxic potencies of **1a**–**n** in the Molt4/C8, CEM and L1210 screens (Figure 4D–F). Thus, in the future, small groups should be placed in the ortho-position in one of the aryl rings.

**Figure 5:**
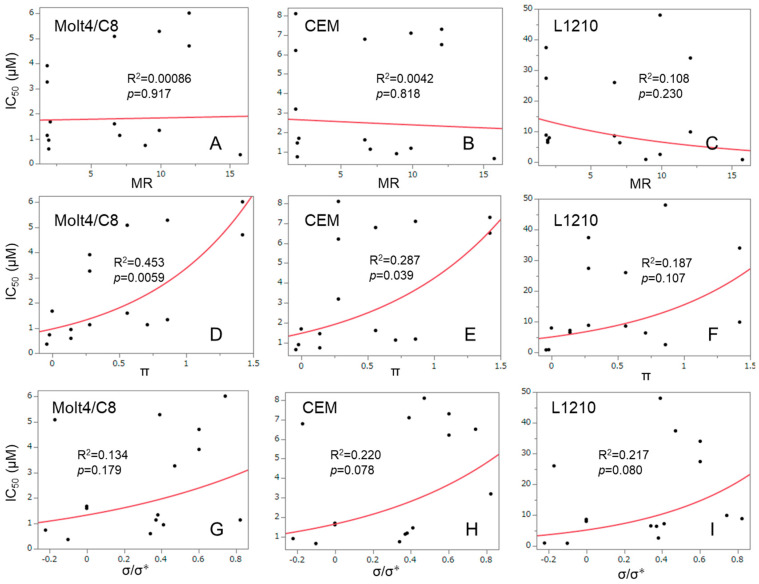
Correlation between the IC_50_ figures of **1a**–**n**/**2a** against Molt4/C8 (**A**,**D**,**G**), CEM (**B**,**E**,**H**) and L1210 (**C**,**F**,**I**) and the MR (**A**–**C**), π (**D**–**F**) or σ/σ* (**G**–**I**). These data are derived from Table 1.

Consideration was given to the other physicochemical properties of the aryl substituents of **1a**–**n** which may influence the bioactivity. Linear and semilogarithmic plots were made between the molar refractivity (MR), Hansch pi (π) and electronic (σ, σ*) constants of the aryl substituents of **1a**–**n**/**2a** and the IC_50_ values generated in the Molt4/C8, CEM and L1210 screens (Figure 5). A positive correlation was noted between the IC_50_ values in the Molt4/C8 screen (R^2^ = 0.453, *p* < 0.01) (D) and the CEM (R^2^ = 0.287, *p* < 0.01) (E) bioassay with the π constants. No other correlations were noted (*p* > 0.078). Thus, in the future, fewer hydrophobic substituents should be placed in the aryl rings.

**Figure 6:**
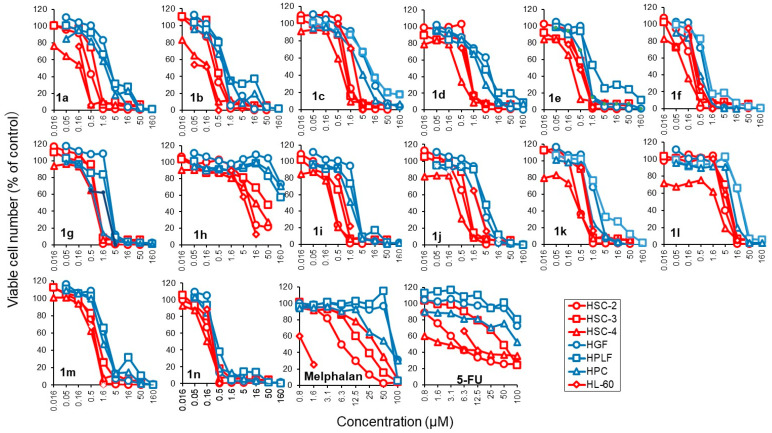
The 3,5-bis(benzylidene)-4-piperidones and melphalan were cytotoxic, while the 5-FU was cytostatic. Malignant (red) and non-malignant (blue) cells were treated for 48 h with the indicated concentrations of samples, and the viable cell number was determined by the MTT method. Each value represents the mean of triplicate determinations. It should be noted that the concentration of compounds (**1a**–**1n**) increases at the ratio of 1:3.162, whereas that of melphalan and 5-FU increases at the ratio of 1:2.

The next phase in the evaluation of the cytotoxic properties of the compounds in series **1** was to find whether they displayed antineoplastic properties to additional cell lines, and also whether they demonstrated greater toxicity towards neoplasms than non-malignant cells. The dienones were screened against HSC-2, HSC-3, HSC-4 and HL-60 neoplastic cell lines (Figure 6), and the biodata generated are displayed in Table 2. The results confirm that, in general, the compounds in this series are potent cytotoxins. Thus, 61% of the CC_50_ values are submicromolar and the average CC_50_ figures are below 1 μM for **1a**,**b**,**d**–**f**,**i**,**k**,**m**,**n**. With the exception of **1h**, the average CC_50_ figures of other members of series **1** are substantially lower than the values for the established anticancer drugs melphalan and 5-fluorouracil. For example, the average CC_50_ figure of **1n** is 58 times lower than the comparable value for melphalan. Represented dose–response curves are shown in Figure 6. Compounds **1a**, **1c**, **1d** and **1h** completely killed the cells (cytotoxic action) rather than providing growth inhibition (cytostatic action). The 5-FU did not completely kill the cells (cytostatic growth inhibition), while melphalan showed cytotoxic action. These patterns were similar regardless of whether the cells were malignant or non-malignant.

The dienones **1a**–**n** were also evaluated against human HGF, HPC and HPLF non-malignant cells, and these results are presented in Table 3. In clinical practice, individual tumors are surrounded by a variety of non-malignant cells. Hence, selectivity index (SI) figures were calculated, in which the potency of the compound towards a specific cell line was compared to the average CC_50_ values of HGF, HPC and HPLF cells. These SI values are portrayed in Table 2. All of the SI figures are above one, and thus the compounds demonstrate greater toxicity to the four neoplasms than to three normal cell lines. This result is encouraging, and the generality of these compounds being tumor-selective cytotoxins should be pursued. Furthermore, the average SI values using HSC-2, HSC-3, HSC-4 and HL-60 cells are 6.69, 4.48, 10.83 and 6.61, respectively (Table 2), indicating that the cell lines vary in their sensitivity to series **1**. This difference in the sensitivities of neoplastic cells to these compounds is an additional indicator that the compounds are not general biocidal agents but have the potential to demonstrate selective toxicity. In the future, this observation of differential sensitivity should be examined further by evaluating representative compounds against additional neoplastic and non-malignant cell lines. Compounds with average SI values greater than five are **1a**–**f**,**i**–**k**,**n**, i.e., in 77% of the dienones.

In order to identify promising lead compounds with both cytotoxic potencies towards neoplastic cells as well as SI values, the generation of potency-selectivity expression (PSE) values was taken into consideration for each of the compounds in series **1**. The PSE is the product of the reciprocal of the average CC_50_ values towards the four neoplastic cell lines (potency) and the average SI figures (selectivity). This value is useful for the treatment of cancer patients, since compounds with high PSE values exhibit high tumor-selective cytotoxicity in small doses. The PSE values are listed in Table 3. The highest PSE figures in excess of 25 were demonstrated by **1a**,**b**,**f**, while the PSE values of **1e**,**i**,**n** were within the range of 10–25. Hence, in developing these compounds, the aryl-substituted pattern found in **1a**,**b**,**e**,**f**,**i**,**n** should be taken into consideration. On the other hand, the 2,6-dichloro (**1h**) and 2,4-dimethyl (**1l**) analogs have very low PSE figures. However, apart from these two compounds, the PSE values of the other members of series **1** are higher than the figure of 1.72 for melphalan.

## 5. Conclusions

A series of 3,5-bis(benzylidene)-4-piperidones, **1a**–**n**, have been prepared in order to find whether, in general, the placement of aryl substituents in the ortho-position increases cytotoxic potencies versus the structural isomers which have groups in the meta- and para-positions. Evaluations of **1a**–**n** against Molt4/C8, CEM and L1210 cells revealed that, in general, the compounds display excellent cytotoxic properties and are either more potent than analogs containing the same aryl substituent, but located in the meta- and para-positions, or are equipotent. Promising compounds from this bioevaluation are **1m** and **1n**, which have submicromolar IC_50_ values towards all three cell lines. Compounds with low θ_A_ values in one of the aryl rings and aryl substituents with small π constants should be prepared in the future development of these compounds.

The evaluation of the compounds in series **1** against additional neoplastic cell lines, as portrayed in Table 2, confirmed that these dienones are potent cytotoxins. They display less toxicity towards three non-malignant cells and, hence, demonstrate tumor-selective toxicity. Taking into consideration both the potency and average SI values, **1a**,**b**,**f** and also **1e**,**i**,**n** are clearly lead molecules, with PSE figures that are substantially higher than melphalan.

## Figures and Tables

**Table 2:** Evaluation of **1a**–**n** against some human tumor cells.

Compound	CC_50_ (µM) ^a^									
	HSC-2	SI ^b^	HSC-3	SI ^b^	HSC-4	SI ^b^	HL-60	SI ^b^	Ave. CC_50_	Ave. SI
**1a**	0.57 ± 0.24	8.12	0.89 ± 0.08	5.20	0.18 ± 0.08	25.7	0.29 ± 0.02	16.0	0.48	13.8
**1b**	0.41 ± 0.12	5.81	0.47 ± 0.06	5.06	0.19 ± 0.08	12.5	0.17 ± 0.10	14.0	0.31	9.34
**1c**	1.27 ± 0.21	6.32	1.06 ± 0.16	7.58	0.72 ± 0.04	11.2	2.60 ± 0.03	3.09	1.41	7.05
**1d**	1.17 ± 0.06	5.28	1.06 ± 0.08	5.83	0.41 ± 0.14	15.1	1.00 ± 0.27	6.18	0.91	8.10
**1e**	0.53 ± 0.17	4.74	0.67 ± 0.29	3.75	0.25 ± 0.10	10.0	0.60 ± 0.28	4.18	0.51	5.67
**1f**	0.24 ± 0.07	8.00	0.35 ± 0.05	5.49	0.12 ± 0.10	16.0	0.42 ± 0.10	4.57	0.28	8.52
**1g**	0.95 ± 0.13	4.04	1.10 ± 0.10	3.49	0.75 ± 0.20	5.12	2.60 ± 0.56	1.48	1.35	3.53
**1h**	10.67 ± 0.8	>14.8	45.33 ± 7.23	>3.52	44.67 ± 1.53	>3.58	7.00 ± 2.90	>22.8	26.9	>11.2
**1i**	0.37 ± 0.07	11.1	0.79 ± 0.06	5.18	0.32 ± 0.02	12.8	1.20 ± 0.10	3.41	0.67	8.12
**1j**	0.81 ± 0.42	6.48	1.06 ± 0.06	4.95	0.38 ± 0.08	13.8	2.60 ± 0.04	2.02	1.21	6.81
**1k**	0.62 ± 0.42	6.23	1.04 ± 0.06	3.71	0.45 ± 0.30	8.58	1.10 ± 0.21	3.51	0.80	5.51
**1l**	4.47 ± 0.21	3.00	8.17 ± 2.16	1.64	2.80 ± 1.39	4.79	6.70 ± 2.30	2.00	5.54	2.86
**1m**	0.87 ± 0.10	4.16	1.13 ± 0.12	3.20	0.74 ± 0.14	4.89	0.74 ± 0.18	4.89	0.87	4.29
**1n**	0.24 ± 0.02	5.63	0.33 ± 0.01	4.09	0.18 ± 0.08	7.50	0.31 ± 0.01	4.36	0.27	5.40
**(Average)**		6.69		4.48		10.83		6.61		
Melphalan	6.20 ± 0.32	13.9	19.00 ± 0.58	4.54	36.00 ± 1.7	2.40	1.00 ± 0.04	86.3	15.6	26.8
5-Fluorouracil	4.90 ± 0.91	>20.4	47.00 ± 7.20	>2.13	2.50 ± 0.25	>40	10.00 ± 1.1	>10.0	16.1	>18.1

^a^ The CC_50_ value is the concentration of the compound required to kill 50% of the cells. ^b^ The letters SI refer to the selectivity index, which is the quotient of the average CC_50_ value towards non-malignant cells and the CC_50_ figure generated against a specific neoplastic cell line.

**Table 3:** Evaluation of **1a**–**n** against some non-malignant cells.

Compound	CC_50_ (µM) ^a^				PSE ^b^
	HGF	HPC	HPLF	Ave. CC_50_	
**1a**	5.57 ± 2.37	3.83 ± 0.55	4.50 ± 0.44	4.63	28.8
**1b**	2.21 ± 0.09	2.33 ± 0.55	2.60 ± 0.17	2.38	30.1
**1c**	8.73 ± 0.64	5.56 ± 0.05	9.80 ± 1.04	8.03	5.00
**1d**	7.70 ± 0.50	4.60 ± 0.44	6.23 ± 0.74	6.18	8.90
**1e**	2.47 ± 0.11	1.50 ± 0.20	3.57 ± 0.71	2.51	11.1
**1f**	1.95 ± 0.46	1.43 ± 0.38	2.37 ± 0.12	1.92	30.4
**1g**	5.03 ± 0.32	1.97 ± 0.50	4.53 ± 0.23	3.84	2.62
**1h**	>160	>160	>160	>160	>0.42
**1i**	4.68 ± 0.21	3.40 ± 0.62	4.20 ± 0.26	4.09	12.1
**1j**	5.40 ± 0.66	3.93 ± 1.00	6.43 ± 1.12	5.25	5.63
**1k**	4.00 ± 1.13	2.55 ± 0.09	5.02 ± 0.10	3.86	6.89
**1l**	15.03 ± 3.86	10.10 ± 1.39	15.00 ± 3.00	13.4	0.52
**1m**	3.80 ± 0.78	3.13 ± 0.42	3.93 ± 1.08	3.62	4.93
**1n**	1.36 ± 0.13	1.19 ± 0.02	1.50 ± 0.40	1.35	20.0
Melphalan	96.00 ± 6.40	83.00 ± 4.50	80.00 ± 0.58	86.3	1.72
5-Fluorouracil	>100	>100	>100	>100	>1.12

^a^ The CC_50_ value is the concentration of the compound required to kill 50% of the cells. ^b^ The potency-selectivity expression (PSE) is the product of the reciprocal of the average CC_50_ value towards four neoplastic cell lines and the average SI values.

## Data Availability

Data are contained within the article.

## Supplementary Materials

The following supporting information can be downloaded at: https://www.mdpi.com/article/10.3390/medicines11080019/s1, ^1^H NMR spectra of the compounds **1a**–**n**.
