# Supplementary material for: Does Ortho-Substitution Enhance Cytotoxic Potencies in a Series of 3,5-Bis(benzylidene)-4-piperidones?
Source: Medicines (Basel). 2024 Oct 30;11(8):19. doi: 10.3390/medicines11080019 (PMC11587156; doi:10.3390/medicines11080019)
Supplement: Supplementary file 1 [file medicines-11-00019-s001.zip › medicines-3144668-supplementary.pdf]

SUPPLEMENTAL SECTION

Does ortho substitution enhance cytotoxic potencies in a series of 3,5-bis(benzylidene)-4-piperidones?

Subhas S. Karki[1,5], Umashankar Das[1], Jan Balzarini[2], Erik De Clercq[2], Hiroshi Sakagami[3], Yoshihiro Uesawa[4], Praveen K. Roayapalley[1,*] and Jonathan R. Dimmock[1]

[1] College of Pharmacy and Nutrition, University of Saskatchewan, Saskatoon, Saskatchewan S7N 5C9, Canada

[2] Rega Institute of Medical Research, Katholieke Universiteit Leuven, 3000 Leuven, Belgium

[3] Mekei University Research Institute of Odontology (M-RIO), Saitama 350-0238, Japan

[4] Department of Medical Molecular Informatics, Meiji Pharmaceutical University, Tokyo 204-858, Japan

[5] Department of Pharmaceutical Chemistry, KLE College of Pharmacy-Bengaluru, (A Constituent unit of KAHER-Belagavi), Karnataka 560010, India

*Correspondence: rpraveen.sp@usask.ca ; Tel.: (+1) 3067154217

Contents:

^{1}H NMR spectra of the compounds **1a-1n**……………………………………………. 2 - 15

128C

3,5-bis(2-Fluorobenzylidene)-4-piperidone (**1a**)

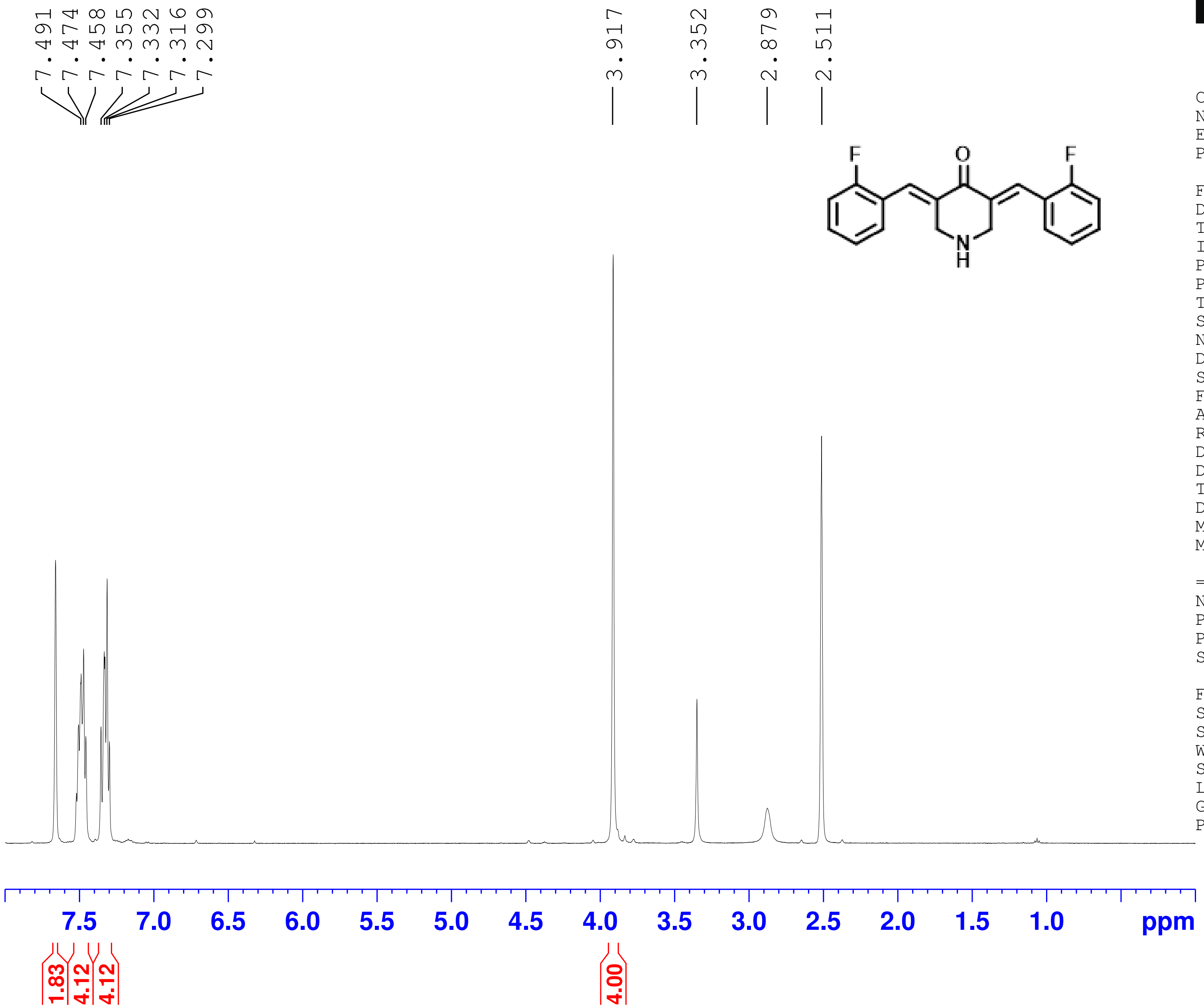


```
Current Data Parameters
NAME              SK-01-128C
EXPNO                      1
PROCNO                     1

F2 - Acquisition Parameters
Date_               20071221
Time                    3.45
INSTRUM                spect
PROBHD       5 mm PABBO BB-
PULPROG                 zg30
TD                     65536
SOLVENT                CDCl3
NS                        16
DS                         2
SWH                10330.578 Hz
FIDRES              0.157632 Hz
AQ                 3.1719425 sec
RG                     203.2
DW                    48.400 usec
DE                      6.00 usec
TE                         0 K
D1                1.00000000 sec
MCREST                     0 sec
MCWRK             0.01500000 sec

======== CHANNEL f1 ========
NUC1                      1H
P1                     12.00 usec
PL1                    -2.00 dB
SFO1             500.2830894 MHz

F2 - Processing parameters
SI                     32768
SF               500.2800000 MHz
WDW                       EM
SSB                        0
LB                      0.30 Hz
GB                         0
PC                      1.00
```

128B

3,5-bis(3-Fluorobenzylidene)-4-piperidone (**1b**)

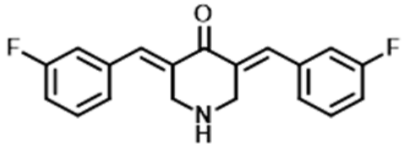

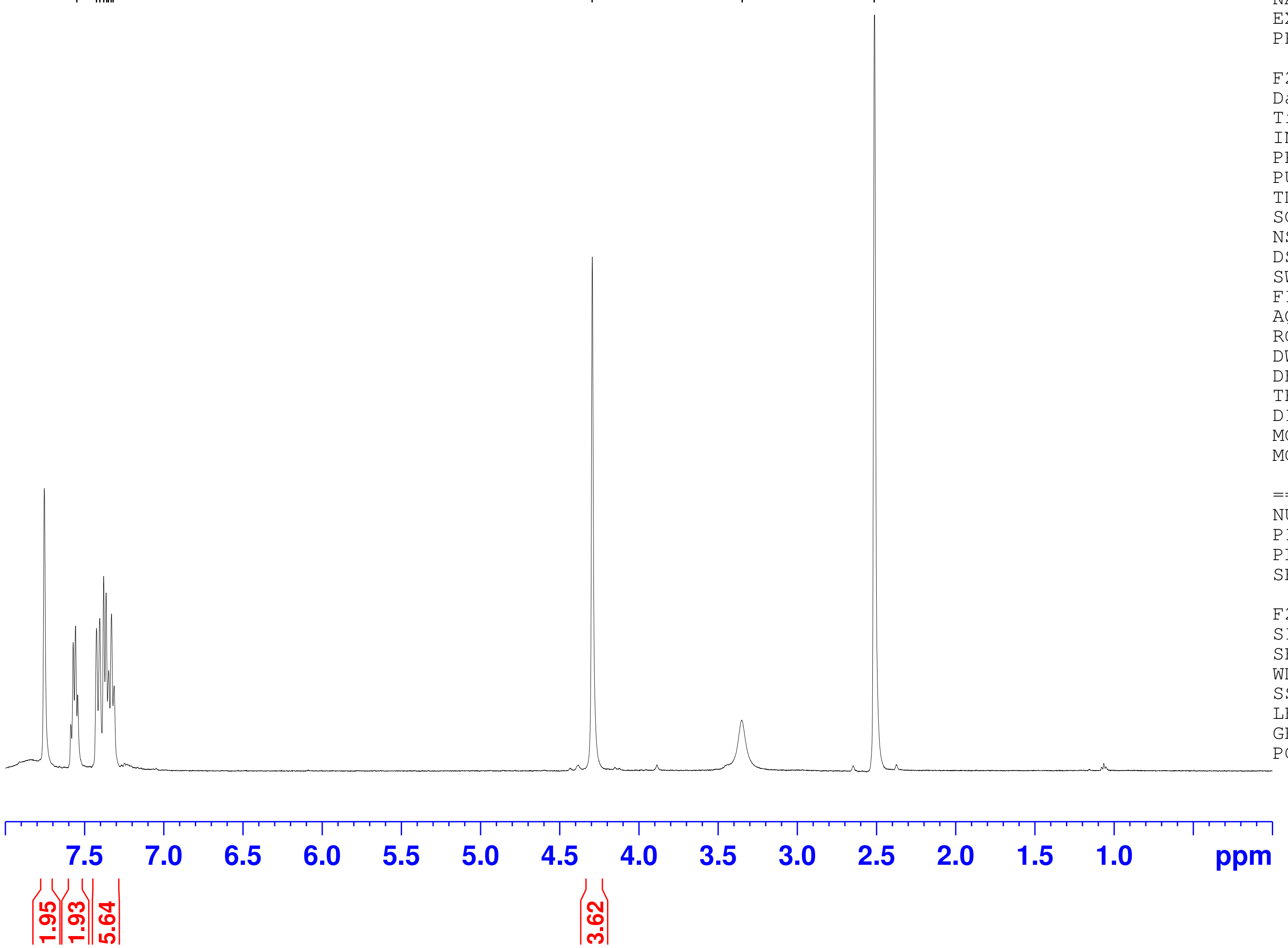

BRUKER

Current Data Parameters

NAME	SK-01-128B
EXPNO	1
PROCNO	1

F2 - Acquisition Parameters

Date_	20071228
Time	21.19
INSTRUM	spect
PROBHD	5 mm PABBO BB-
PULPROG	zg30
TD	65536
SOLVENT	CDCl3
NS	16
DS	2
SWH	10330.578 Hz
FIDRES	0.157632 Hz
AQ	3.1719425 sec
RG	228.1
DW	48.400 usec
DE	6.00 usec
TE	0 K
D1	1.00000000 sec
MCREST	0 sec
MCWRK	0.01500000 sec

======== CHANNEL f1 ========

NUC1	1H
P1	12.00 usec
PL1	-2.00 dB
SFO1	500.2830894 MHz

F2 - Processing parameters

SI	32768
SF	500.2800000 MHz
WDW	EM
SSB	0
LB	0.30 Hz
GB	0
PC	1.00

131C

3,5-bis(2,4-Difluorobenzylidene)-4-piperidone (**1c**)

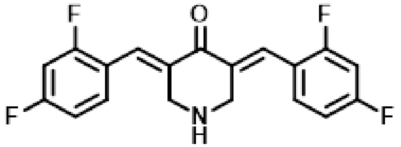

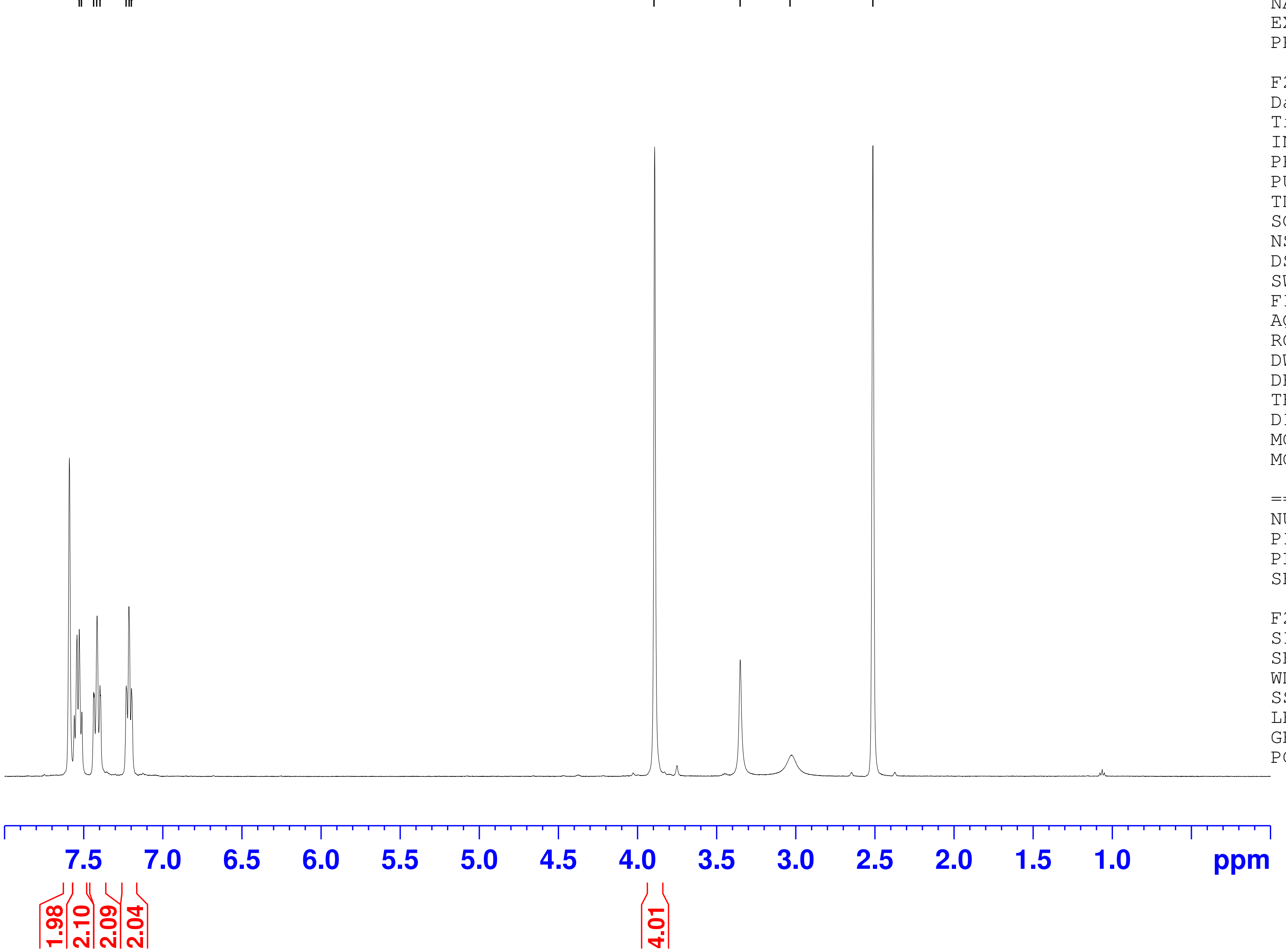


```
Current Data Parameters
NAME           SK-01-131C
EXPNO                   1
PROCNO                  1

F2 - Acquisition Parameters
Date_            20080104
Time                22.29
INSTRUM             spect
PROBHD     5 mm PABBO BB-
PULPROG              zg30
TD                  65536
SOLVENT             CDCl3
NS                     16
DS                      2
SWH             10330.578 Hz
FIDRES           0.157632 Hz
AQ              3.1719425 sec
RG                  203.2
DW                 48.400 usec
DE                   6.00 usec
TE                      0 K
D1             1.00000000 sec
MCREST                  0 sec
MCWRK          0.01500000 sec

======== CHANNEL f1 ========
NUC1                   1H
P1                  12.00 usec
PL1                 -2.00 dB
SFO1          500.2830894 MHz

F2 - Processing parameters
SI                  32768
SF            500.2800000 MHz
WDW                    EM
SSB                     0
LB                   0.30 Hz
GB                      0
PC                   1.00
```

132C

3,5-bis(2,5-Difluorobenzylidene)-4-piperidone (**1d**)

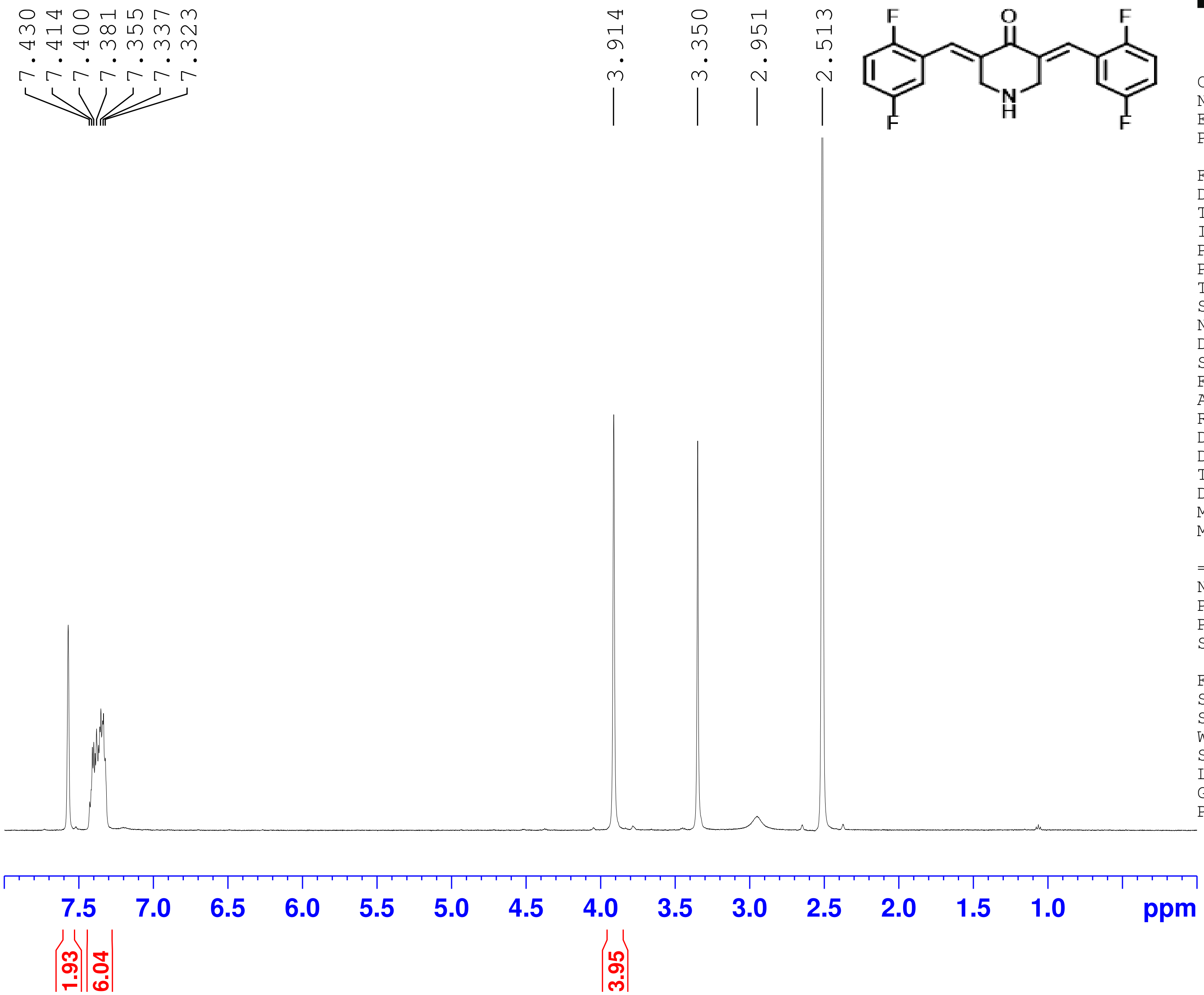

Current Data Parameters
NAME SK-01-132C
EXPNO 1
PROCNO 1

F2 - Acquisition Parameters
Date_ 20080104
Time 22.21
INSTRUM spect
PROBHD 5 mm PABBO BB-
PULPROG zg30
TD 65536
SOLVENT CDCl3
NS 16
DS 2
SWH 10330.578 Hz
FIDRES 0.157632 Hz
AQ 3.1719425 sec
RG 228.1
DW 48.400 usec
DE 6.00 usec
TE 0 K
D1 1.00000000 sec
MCREST 0 sec
MCWRK 0.01500000 sec

======== CHANNEL f1 ========
NUC1 1H
P1 12.00 usec
PL1 -2.00 dB
SFO1 500.2830894 MHz

F2 - Processing parameters
SI 32768
SF 500.2800000 MHz
WDW EM
SSB 0
LB 0.30 Hz
GB 0
PC 1.00

131A

3,5-bis(2,6-Difluorobenzylidene)-4-piperidone (**1e**)

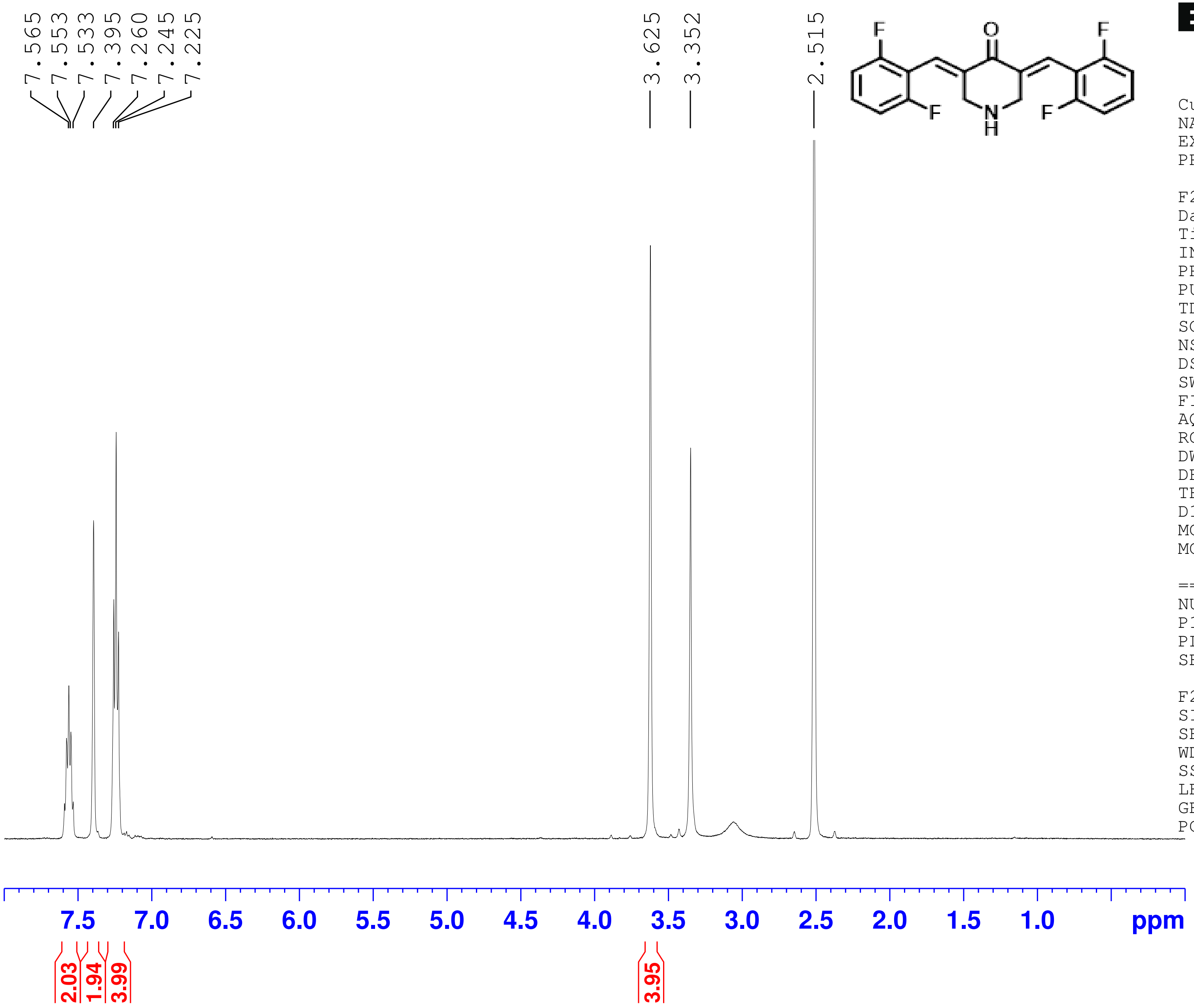


```
Current Data Parameters
NAME          SK-01-131A
EXPNO                  1
PROCNO                 1

F2 - Acquisition Parameters
Date_           20080103
Time               21.14
INSTRUM            spect
PROBHD   5 mm PABBO BB-
PULPROG             zg30
TD                 65536
SOLVENT            CDCl3
NS                    16
DS                     2
SWH            10330.578 Hz
FIDRES          0.157632 Hz
AQ             3.1719425 sec
RG                 228.1
DW                48.400 usec
DE                  6.00 usec
TE                     0 K
D1            1.00000000 sec
MCREST                 0 sec
MCWRK         0.01500000 sec

======== CHANNEL f1 ========
NUC1                  1H
P1                 12.00 usec
PL1                -2.00 dB
SFO1         500.2830894 MHz

F2 - Processing parameters
SI                 32768
SF           500.2800000 MHz
WDW                   EM
SSB                    0
LB                  0.30 Hz
GB                     0
PC                  1.00
```

133B

3,5-bis(2-Chlorobenzylidene)-4-piperidone (**1f**)

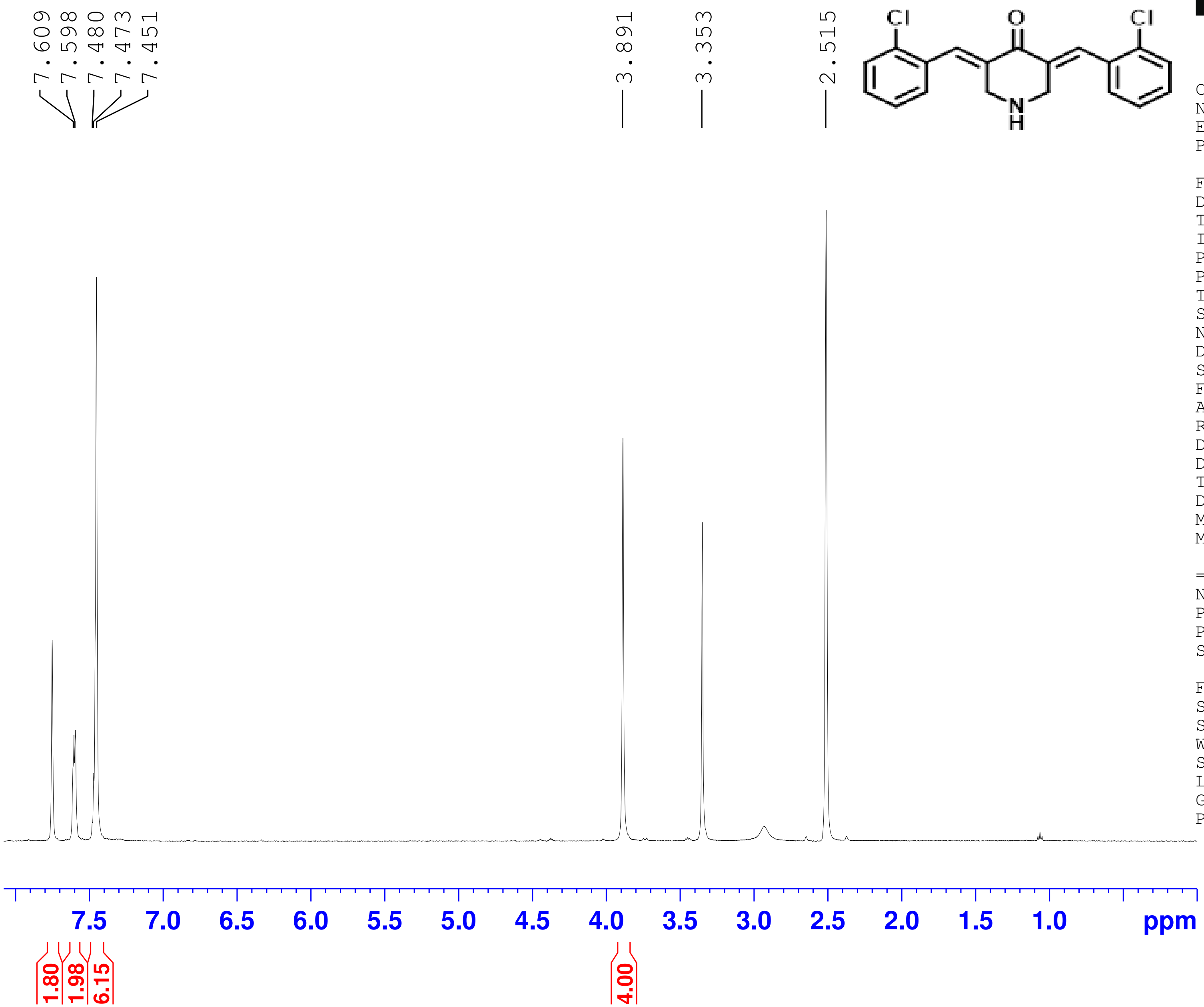

Current Data Parameters

NAME	SK-01-133B
EXPNO	1
PROCNO	1

F2 - Acquisition Parameters

Date_	20080110
Time	22.38
INSTRUM	spect
PROBHD	5 mm PABBO BB-
PULPROG	zg30
TD	65536
SOLVENT	CDCl3
NS	16
DS	2
SWH	10330.578 Hz
FIDRES	0.157632 Hz
AQ	3.1719425 sec
RG	228.1
DW	48.400 usec
DE	6.00 usec
TE	0 K
D1	1.00000000 sec
MCREST	0 sec
MCWRK	0.01500000 sec

======== CHANNEL f1 ========

NUC1	1H
P1	12.00 usec
PL1	-2.00 dB
SFO1	500.2830894 MHz

F2 - Processing parameters

SI	32768
SF	500.2800000 MHz
WDW	EM
SSB	0
LB	0.30 Hz
GB	0
PC	1.00

132A

3,5-bis(2,4-Dichlorobenzylidene)-4-piperidone (**1g**)

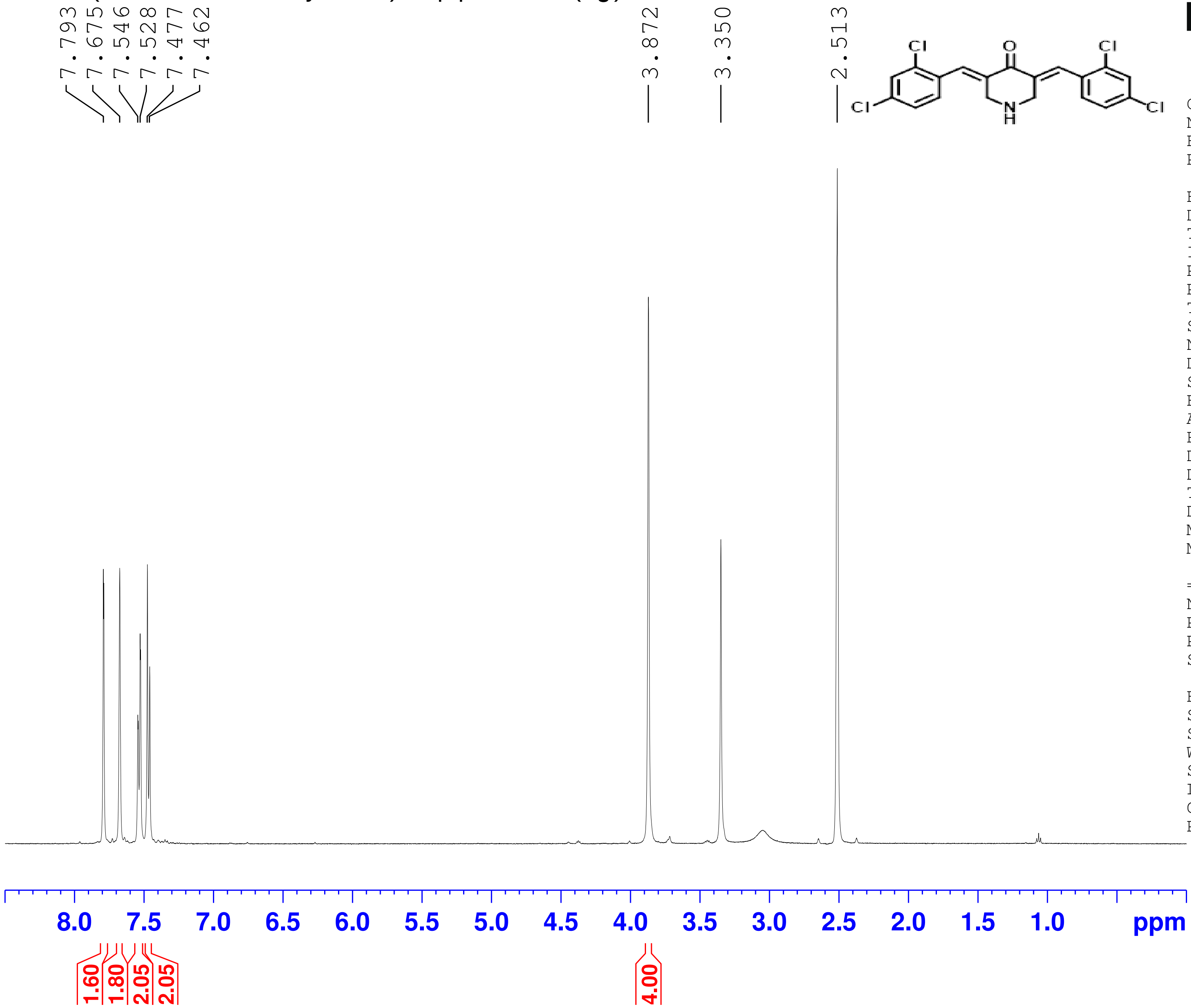

BRUKER

Current Data Parameters

NAME	SK-01-132A
EXPNO	1
PROCNO	1

F2 - Acquisition Parameters

Date_	20080103
Time	21.21
INSTRUM	spect
PROBHD	5 mm PABBO BB-
PULPROG	zg30
TD	65536
SOLVENT	CDCl3
NS	16
DS	2
SWH	10330.578 Hz
FIDRES	0.157632 Hz
AQ	3.1719425 sec
RG	228.1
DW	48.400 usec
DE	6.00 usec
TE	0 K
D1	1.00000000 sec
MCREST	0 sec
MCWRK	0.01500000 sec

======== CHANNEL f1 ========

NUC1	1H
P1	12.00 usec
PL1	-2.00 dB
SFO1	500.2830894 MHz

F2 - Processing parameters

SI	32768
SF	500.2800000 MHz
WDW	EM
SSB	0
LB	0.30 Hz
GB	0
PC	1.00

131B

3,5-bis(2,6-Dichlorobenzylidene)-4-piperidone (1h)

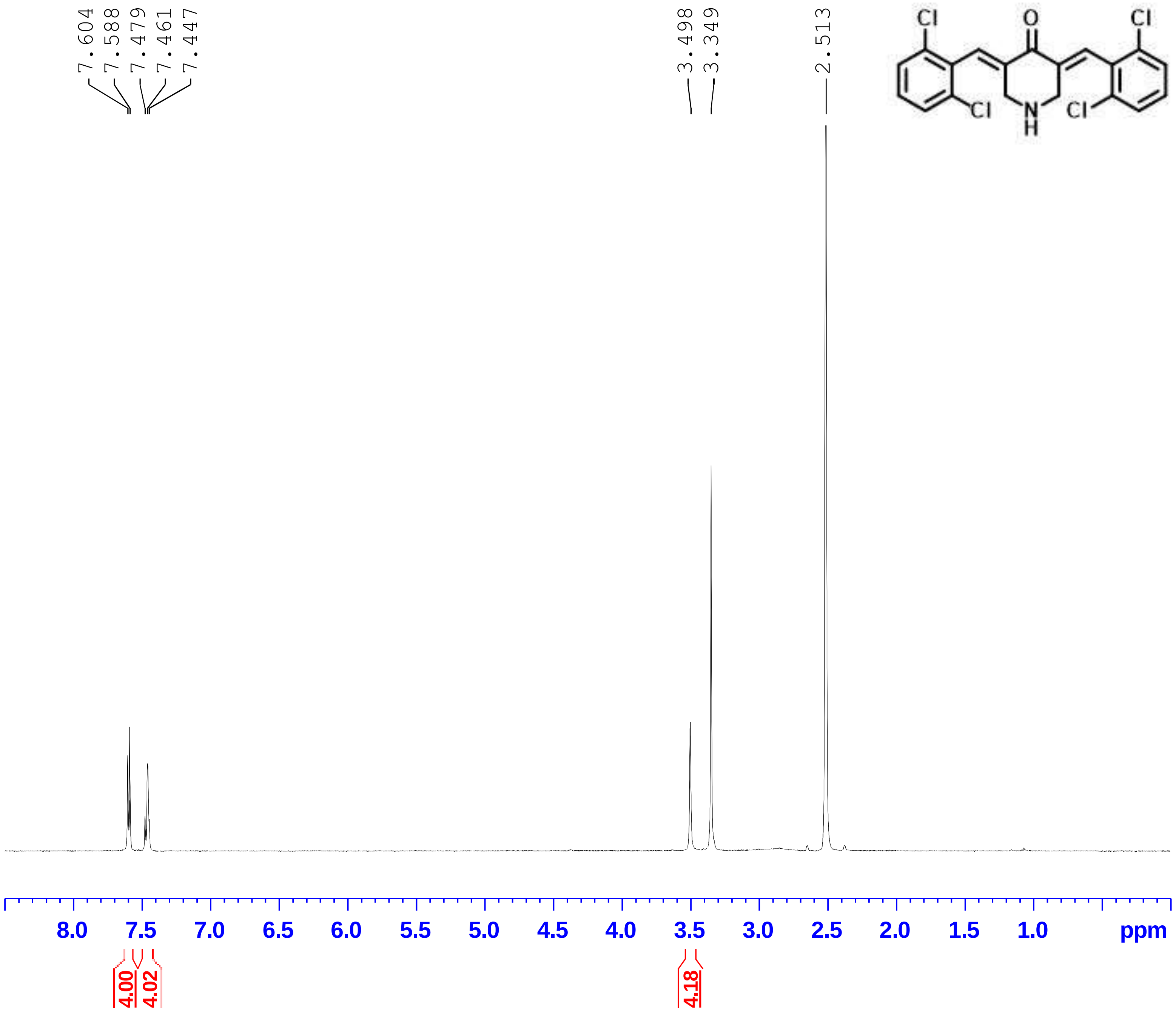

Current Data Parameters
NAME SK-01-131B_test
EXPNO 1
PROCNO 1

F2 - Acquisition Parameters
Date_ 20080128
Time 10.45
INSTRUM spect
PROBHD 5 mm PABBO BB-
PULPROG zg30
TD 65536
SOLVENT D2O
NS 16
DS 2
SWH 10330.578 Hz
FIDRES 0.157632 Hz
AQ 3.1719425 sec
RG 203.2
DW 48.400 usec
DE 6.00 usec
TE 0 K
D1 1.00000000 sec
MCREST 0 sec
MCWRK 0.01500000 sec

======== CHANNEL f1 ========
NUC1 1H
P1 12.00 usec
PL1 -2.00 dB
SFO1 500.2830894 MHz

F2 - Processing parameters
SI 32768
SF 500.2800000 MHz
WDW EM
SSB 0
LB 0.30 Hz
GB 0
PC 1.00

126A

3,5-bis(2-Bromobenzylidene)-4-piperidone (**1i**)

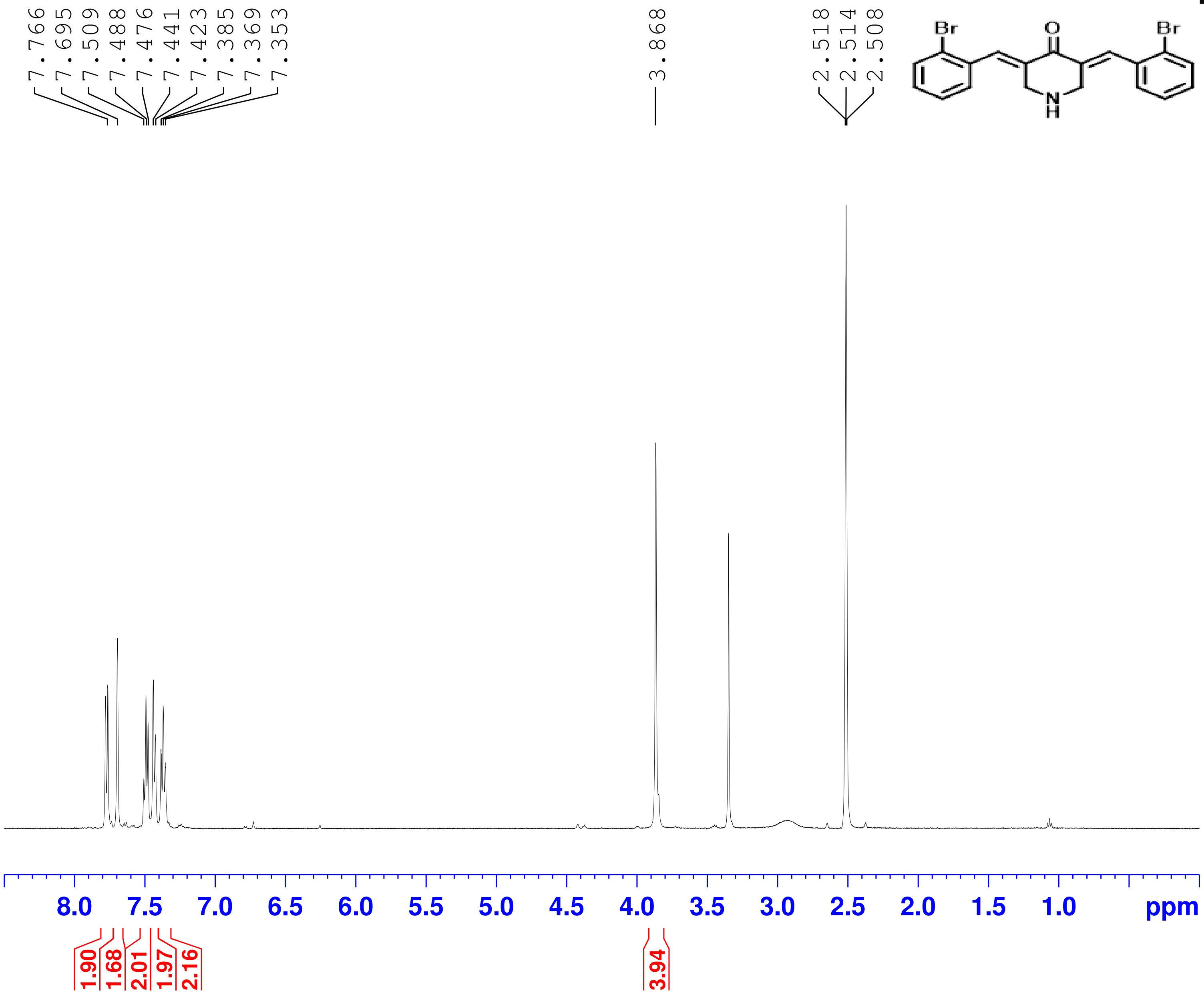

BRUKER

```
Current Data Parameters
NAME             SK-01-126A
EXPNO                     1
PROCNO                    1

F2 - Acquisition Parameters
Date_              20071221
Time                   3.25
INSTRUM               spect
PROBHD     5 mm PABBO BB-
PULPROG                zg30
TD                    65536
SOLVENT               CDCl3
NS                       16
DS                        2
SWH             10330.578 Hz
FIDRES           0.157632 Hz
AQ              3.1719425 sec
RG                    228.1
DW                   48.400 usec
DE                     6.00 usec
TE                        0 K
D1             1.00000000 sec
MCREST                    0 sec
MCWRK          0.01500000 sec

======== CHANNEL f1 ========
NUC1                     1H
P1                    12.00 usec
PL1                   -2.00 dB
SFO1            500.2830894 MHz

F2 - Processing parameters
SI                    32768
SF              500.2800000 MHz
WDW                      EM
SSB                       0
LB                     0.30 Hz
GB                        0
PC                     1.00
```

132B

3,5-bis(3-Bromobenzylidene)-4-piperidone (**1j**)

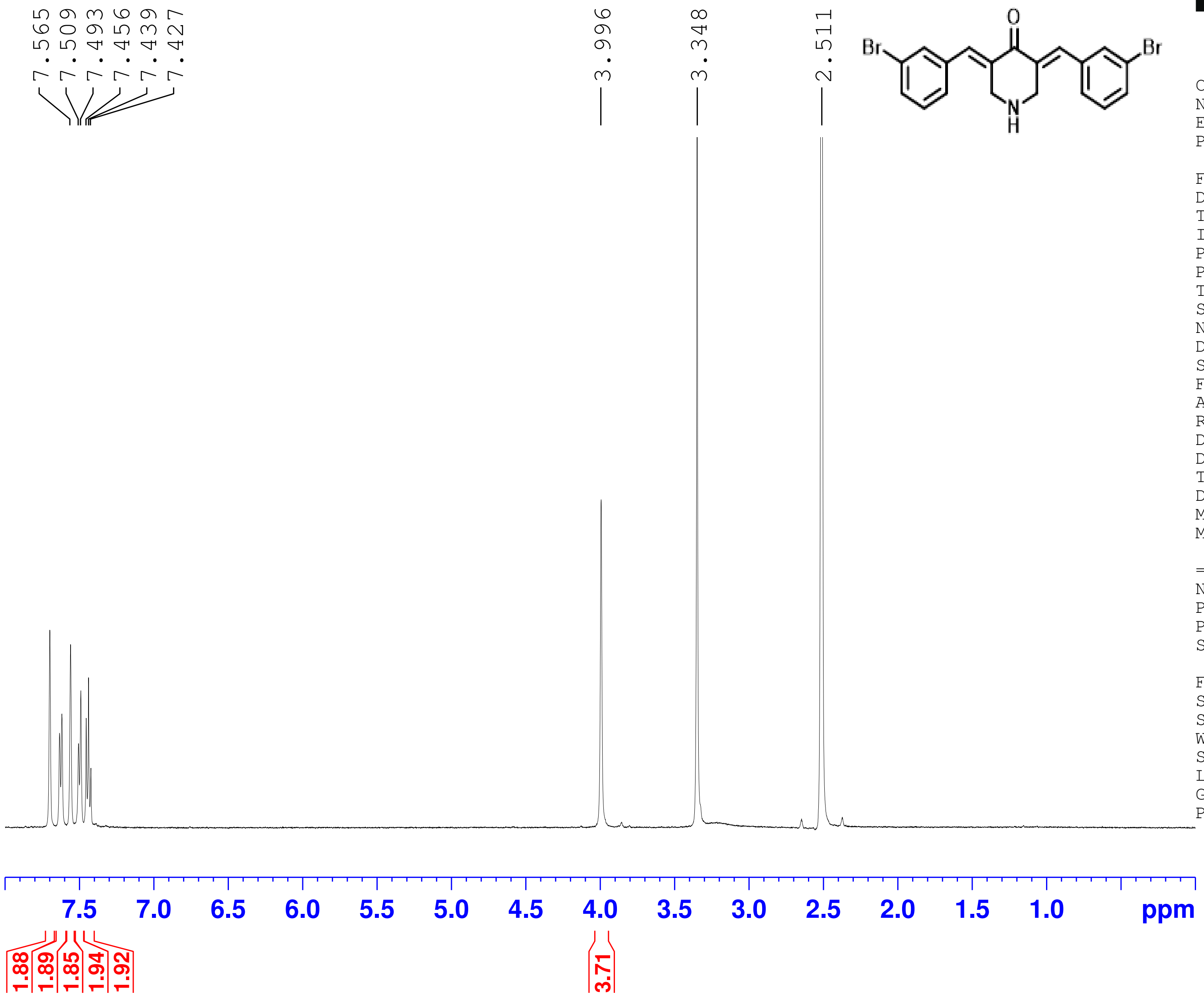


```
Current Data Parameters
NAME          SK-01-132B
EXPNO                  1
PROCNO                 1

F2 - Acquisition Parameters
Date_           20080103
Time               21.29
INSTRUM            spect
PROBHD     5 mm PABBO BB-
PULPROG             zg30
TD                 65536
SOLVENT            CDCl3
NS                    16
DS                     2
SWH            10330.578 Hz
FIDRES          0.157632 Hz
AQ             3.1719425 sec
RG                 228.1
DW                48.400 usec
DE                  6.00 usec
TE                     0 K
D1            1.00000000 sec
MCREST                 0 sec
MCWRK         0.01500000 sec

======== CHANNEL f1 ========
NUC1                  1H
P1                 12.00 usec
PL1                -2.00 dB
SFO1         500.2830894 MHz

F2 - Processing parameters
SI                 32768
SF           500.2800000 MHz
WDW                   EM
SSB                    0
LB                  0.30 Hz
GB                     0
PC                  1.00
```

127B

3,5-bis(2-Methylbenzylidene)-4-piperidone (**1k**)

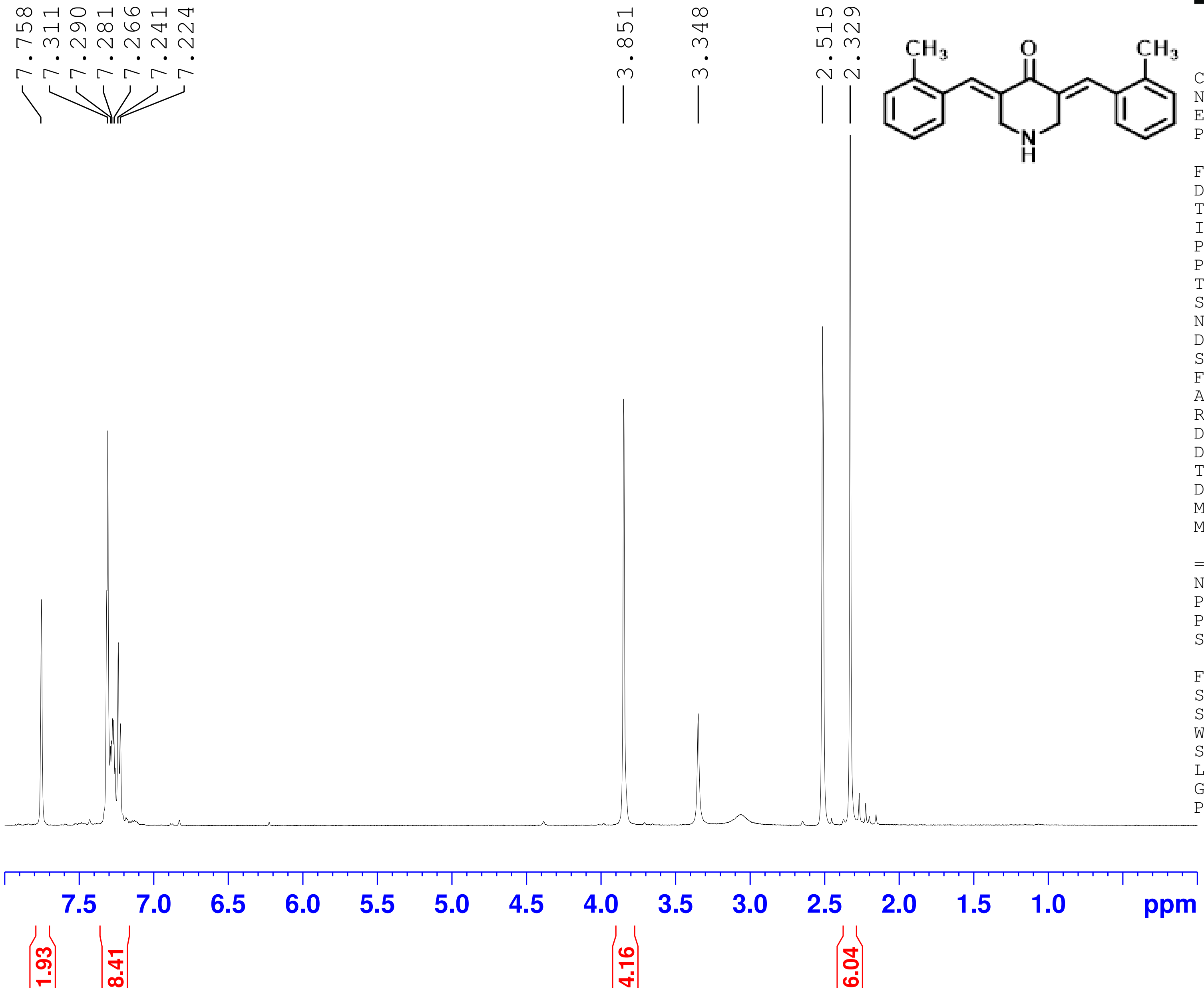


```
Current Data Parameters
NAME              SK-01-127B
EXPNO                      1
PROCNO                     1

F2 - Acquisition Parameters
Date_               20071221
Time                    3.38
INSTRUM                spect
PROBHD       5 mm PABBO BB-
PULPROG                 zg30
TD                     65536
SOLVENT                CDCl3
NS                        16
DS                         2
SWH               10330.578 Hz
FIDRES             0.157632 Hz
AQ                3.1719425 sec
RG                     203.2
DW                    48.400 usec
DE                      6.00 usec
TE                         0 K
D1               1.00000000 sec
MCREST                     0 sec
MCWRK            0.01500000 sec

======== CHANNEL f1 ========
NUC1                      1H
P1                     12.00 usec
PL1                    -2.00 dB
SFO1             500.2830894 MHz

F2 - Processing parameters
SI                     32768
SF               500.2800000 MHz
WDW                       EM
SSB                        0
LB                      0.30 Hz
GB                         0
PC                      1.00
```

132D

3,5-bis(2,4-Dimethylbenzylidene)-4-piperidone (**1l**)

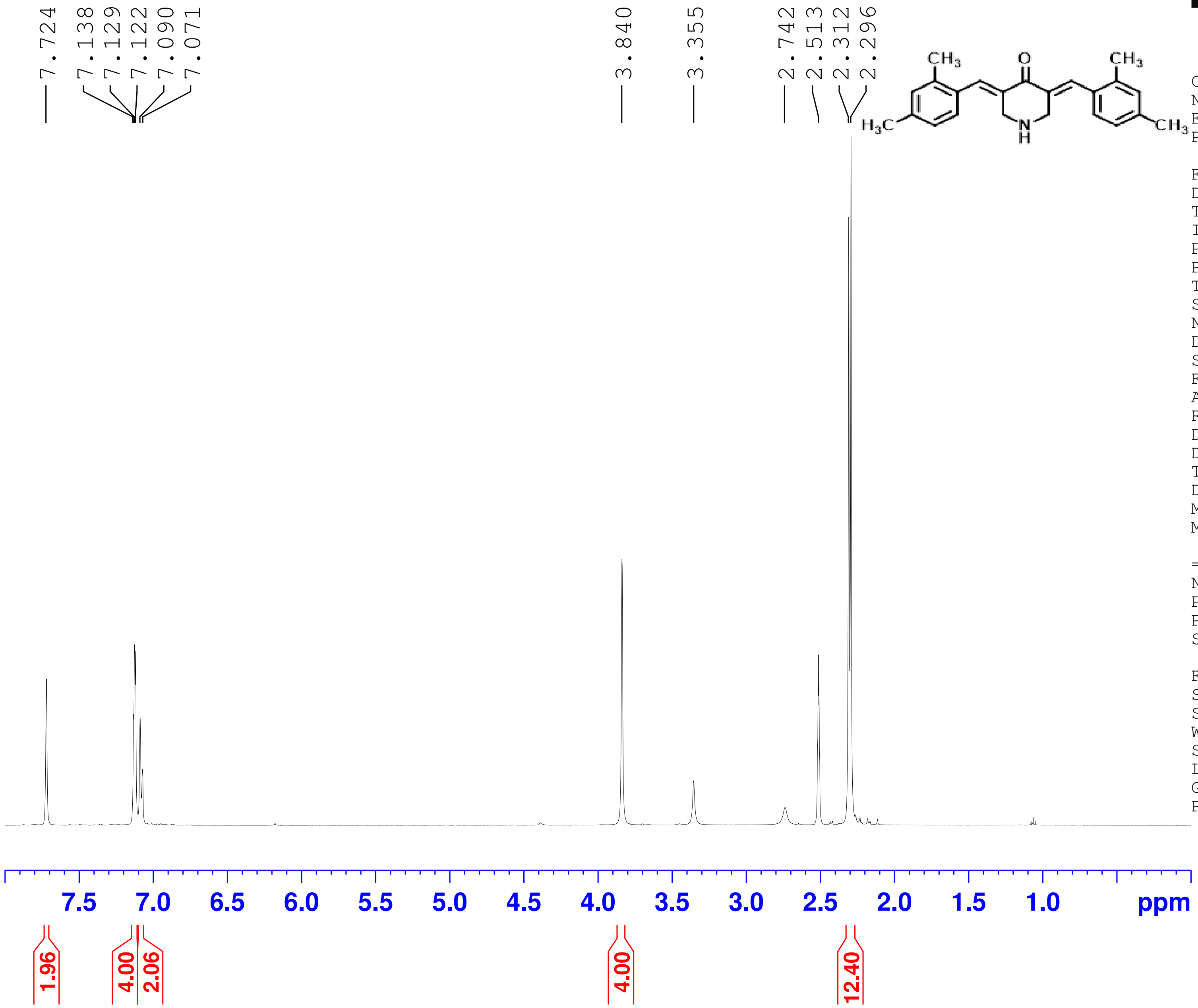

Current Data Parameters
NAME SK-01-132D
EXPNO 1
PROCNO 1

F2 - Acquisition Parameters
Date_ 20080124
Time 0.56
INSTRUM spect
PROBHD 5 mm PABBO BB-
PULPROG zg30
TD 65536
SOLVENT DMSO
NS 32
DS 2
SWH 10330.578 Hz
FIDRES 0.157632 Hz
AQ 3.1719425 sec
RG 143.7
DW 48.400 usec
DE 6.00 usec
TE 0 K
D1 1.00000000 sec
MCREST 0 sec
MCWRK 0.01500000 sec

======== CHANNEL f1 ========
NUC1 1H
P1 12.00 usec
PL1 -2.00 dB
SFO1 500.2830894 MHz

F2 - Processing parameters
SI 32768
SF 500.2800000 MHz
WDW EM
SSB 0
LB 0.30 Hz
GB 0
PC 1.00

126B

3,5-bis(2-Methoxybenzylidene)-4-piperidone (**1m**)

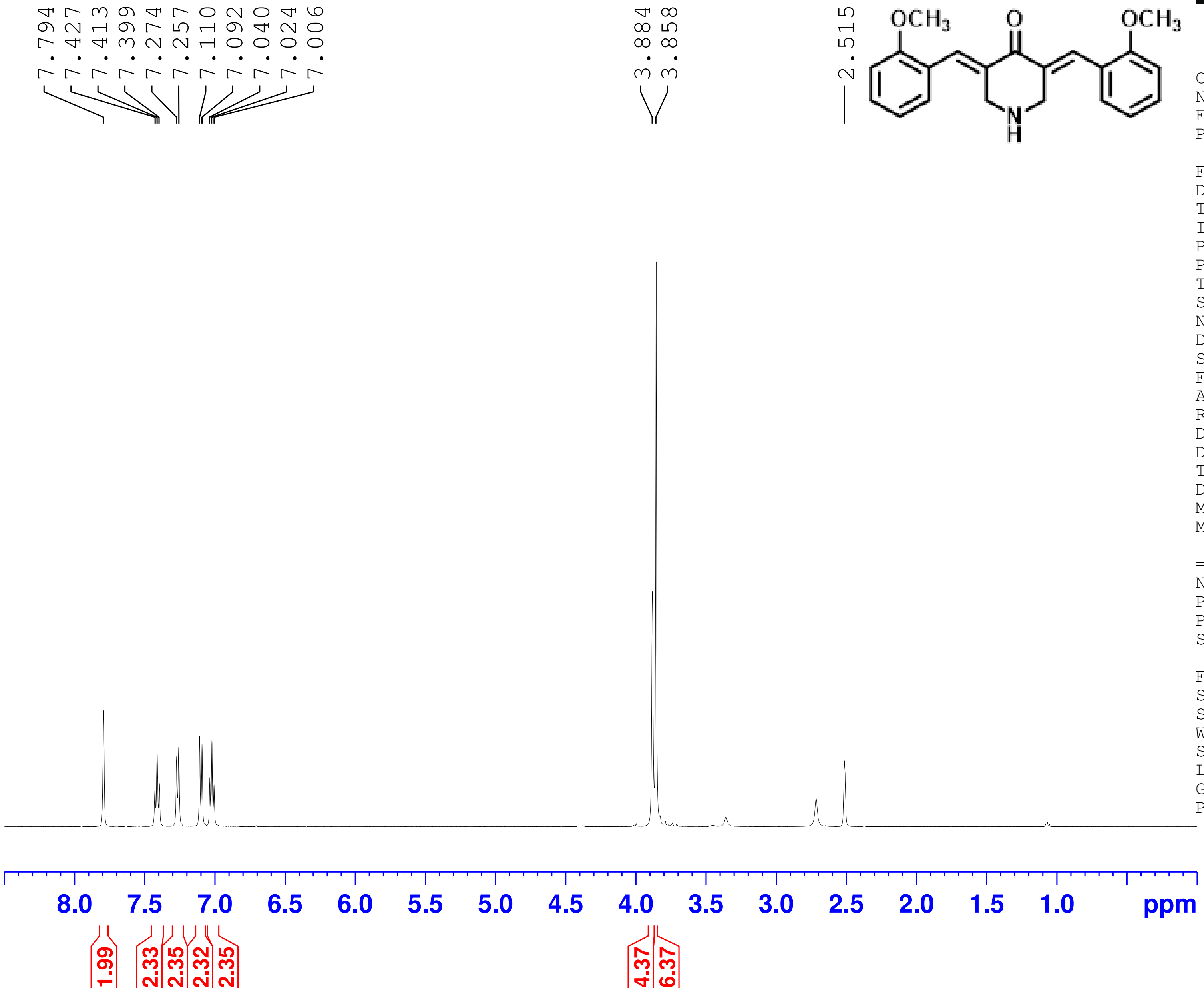

BRUKER

Current Data Parameters
NAME SK-01-126B
EXPNO 1
PROCNO 1

F2 - Acquisition Parameters
Date_ 20071221
Time 3.32
INSTRUM spect
PROBHD 5 mm PABBO BB-
PULPROG zg30
TD 65536
SOLVENT CDCl3
NS 16
DS 2
SWH 10330.578 Hz
FIDRES 0.157632 Hz
AQ 3.1719425 sec
RG 128
DW 48.400 usec
DE 6.00 usec
TE 0 K
D1 1.00000000 sec
MCREST 0 sec
MCWRK 0.01500000 sec

======== CHANNEL f1 ========
NUC1 1H
P1 12.00 usec
PL1 -2.00 dB
SFO1 500.2830894 MHz

F2 - Processing parameters
SI 32768
SF 500.2800000 MHz
WDW EM
SSB 0
LB 0.30 Hz
GB 0
PC 1.00

133A

3,5-bis(2, 3-Dimethoxybenzylidene)-4-piperidone (**1n**)

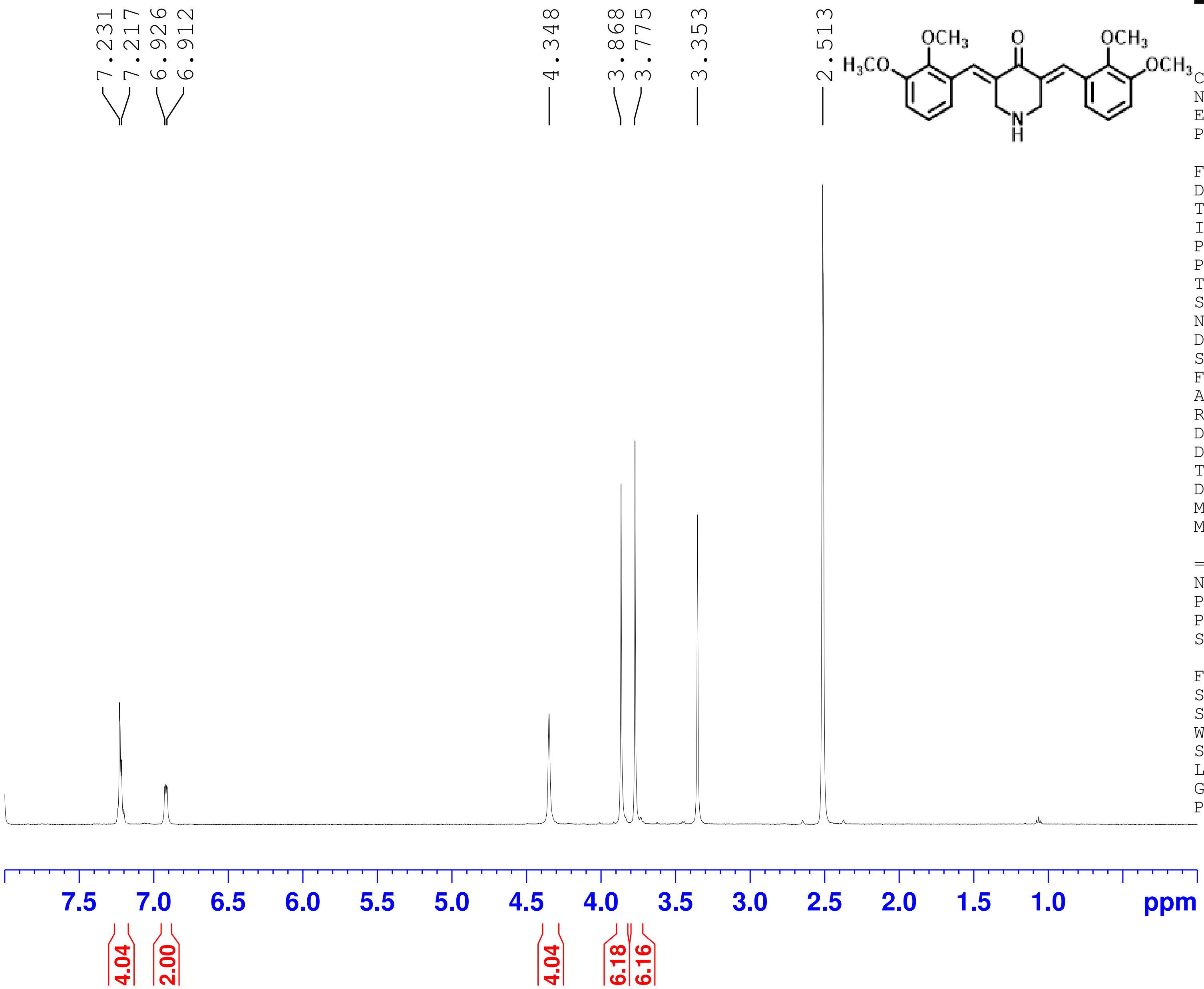

Current Data Parameters
NAME SK-01-133A
EXPNO 1
PROCNO 1

F2 - Acquisition Parameters
Date_ 20080110
Time 22.28
INSTRUM spect
PROBHD 5 mm PABBO BB-
PULPROG zg30
TD 65536
SOLVENT CDCl3
NS 16
DS 2
SWH 10330.578 Hz
FIDRES 0.157632 Hz
AQ 3.1719425 sec
RG 228.1
DW 48.400 usec
DE 6.00 usec
TE 0 K
D1 1.00000000 sec
MCREST 0 sec
MCWRK 0.01500000 sec

======== CHANNEL f1 ========
NUC1 1H
P1 12.00 usec
PL1 -2.00 dB
SFO1 500.2830894 MHz

F2 - Processing parameters
SI 32768
SF 500.2800000 MHz
WDW EM
SSB 0
LB 0.30 Hz
GB 0
PC 1.00
